# A Triangulenum‐Based Biosensor Platform for Mapping Kinase Activity in T Cells

**DOI:** 10.1002/smll.75317

**Published:** 2026-08-20

**Authors:** Carmen Salto‐Giron, Angélica Martín‐Priego, Francisco Fueyo‐González, María Mar Quesada‐Moreno, Federico Movilla, Antonio Reinoso, Laura Espinar‐Barranco, Rafael Salto, Amparo Navarro, Miguel Fribourg, Angel Orte, Juan A. González‐Vera

**Affiliations:** ^1^ Nanoscopy‐UGR Laboratory. Departamento de Fisicoquímica Unidad de Excelencia de Química Aplicada a Biomedicina y Medioambiente Facultad de Farmacia Campus Cartuja Universidad De Granada Granada Spain; ^2^ Department of Pharmacological Sciences Institute for Translational Medicine and Pharmacology Immunology Institute Icahn School of Medicine at Mount Sinai New York New York USA; ^3^ Department of Medicine Translational Transplant Research Center Immunology Institute Icahn School of Medicine at Mount Sinai New York New York USA; ^4^ Departamento De Química Física y Analítica Facultad De Ciencias Experimentales Universidad De Jaén Jaén Spain; ^5^ Departamento De Bioquímica y Biología Molecular II Unidad de Excelencia de Química Aplicada a Biomedicina y Medioambiente Facultad De Farmacia Campus Cartuja Universidad De Granada Granada Spain

**Keywords:** bioimaging, biosensor, DFT, FLIM, Kinase, T Cell, triangulenium

## Abstract

Monitoring protein kinases in their native environment is essential for understanding cellular signaling. This study introduces triangulenium‐based peptide biosensors for detection of the activity of two key protein kinases involved in T cell immune response: Akt1 and Src. The substrate biosensors incorporate a long‐lifetime fluorophore from the triangulenium dye family, which undergoes quenching by photoinduced electron transfer (PET) from different amino acid side groups. Upon kinase‐mediated phosphorylation, the PET quenching is significantly suppressed, increasing both fluorescence intensity and fluorescence lifetime (τ_FL_). This lifetime extension (up to 15 ns) enables the use of fluorescence lifetime imaging microscopy (FLIM) and time‐gated filtering to effectively eliminate cellular autofluorescence. In vitro and in extracto assays confirmed the biosensors' high affinity and specificity for endogenous kinases. FLIM experiments in HeLa cells demonstrated efficient uptake, biocompatibility, and quantitative reporting of endogenous Src and Akt1 activities through robust, lifetime‑based responses selectively modulated by kinase activators and inhibitors. Crucially, the Akt1 biosensor tracked dynamic signaling changes in isolated primary T cells following T cell receptor stimulation using confocal microscopy and flow cytometry. This integrated framework provides a robust, non‐invasive platform for quantitative kinase analysis in living cells, with significant implications for immunological profiling and cancer research.

## Graphical abstract

Novel peptide biosensors based on long‐lifetime triangulenium dyes are developed for the real‐time, non‐invasive mapping of Akt1 and Src kinase activities in living cells. Utilizing FLIM microscopy, these highly specific, biocompatible probes overcome cellular autofluorescence. The platform successfully tracks dynamic, endogenous kinase signaling changes within primary immune cells.

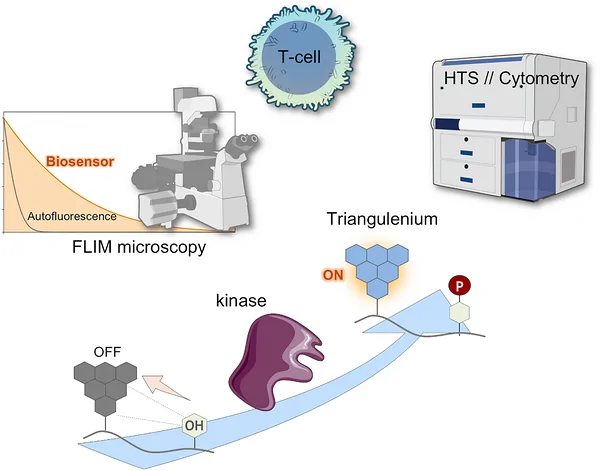

## 1 Introduction

Protein phosphorylation, mediated by protein kinases, is a central mechanism of cellular signal transduction that regulates virtually all aspects of cell physiology. By transferring the γ‐phosphate group from ATP to Ser, Thr, or Tyr residues, protein kinases modulate activation and inhibition of diverse signaling pathways [1]. Dysregulated kinase activity—arising from aberrant expression, mutations, or altered upstream signaling—is implicated in a broad spectrum of diseases, including autoimmune disorders, cancer, and pro‐inflammatory responses such as those associated with COVID‐19 [1, 2, 3, 4].

Recent studies highlight the importance of kinase signaling in regulating the immunological function of T cells [5]. Despite their importance, current methods to monitor kinase activity in T cells remain limited. Conventional assays such as western blotting, flow cytometry with phospho‐specific antibodies, and mass spectrometry provide powerful biochemical information but are destructive, endpoint techniques that lack real‐time and single‐cell resolution. Because these approaches require cell fixation or lysis, they preclude recovery and isolation of viable cells, thereby limiting downstream functional studies. This is particularly restrictive when studying dynamic processes such as metabolic reprogramming, which is tightly integrated with phosphorylation networks and kinase‐driven signaling cascades. As a result, transient or heterogeneous signaling events critical for downstream functional outcomes may be overlooked [6, 7]. Moreover, standard diagnostic tools for assessing kinase dysregulation are often insufficient and typically rely on indirect readouts. These limitations underscore the need for innovative sensing technologies capable of sensitive, rapid, and non‑invasive monitoring of kinase activity in living immune cells.

Luminescence‐based methods offer key advantages for addressing these challenges: they are non‐destructive, highly sensitive, and provide excellent spatial and temporal resolution. In this context, fluorescent biosensors have emerged as promising tools for real‐time kinase activity monitoring. Two major classes of these sensors exist. Genetically encoded biosensors rely on fluorescent protein pairs flanking a kinase substrate and a phosphoamino acid binding domain (PAABD). Phosphorylation induces PAABD binding and triggers a Förster Resonance Energy Transfer (FRET) response; however, such sensors typically exhibit modest FRET efficiency (<50%), limiting their sensitivity [8, 9]. Additionally, their use requires genetic manipulation of the target cells, which can perturb the native cellular environment and signaling architecture. This is particularly problematic in primary human T cells, where genetic modification may alter physiological responses and limit direct applicability for biosensing or diagnostic purposes in clinically relevant samples. Peptide‐based biosensors, by contrast, use fluorophore‐labeled peptide substrates whose optical properties change upon phosphorylation. They offer high modularity and large fluorescence modulation but are commonly restricted to in vitro assays and rely on intensity‐based measurements, which are sensitive to probe concentration, photobleaching, and cellular autofluorescence [8, 9]. Among the various peptide‐based biosensors described in the literature, quenching‐based designs provide a powerful strategy for kinase sensing [10, 11, 12, 13, 14, 15, 16, 17]. Many designs exploit the intrinsic ability of aromatic residues—especially Tyr and Trp—to quench nearby fluorophores through dynamic and static quenching, the latter involving π–π stacking interactions [18, 19]. Phosphorylation disrupts these interactions, producing a fluorescence turn‐on response. Early examples include a pyrene‐labeled Src substrate in which phosphorylation of a Tyr residue led to dye–Tyr unstacking and strong fluorescence enhancement [10]. However, these assays typically rely on fluorescence intensity measurements. While highly sensitive, this approach is susceptible to systematic quantification errors caused by fluorescent interferents commonly present in biological fluids, which can limit its applicability in *in cellulo* assays.

In this regard, fluorescence lifetime imaging microscopy (FLIM) is a powerful alternative for intracellular sensing [20, 21, 22]. FLIM resolves both fluorescence intensity and the fluorescence lifetime (τ_FL_), a parameter independent of fluorophore concentration and excitation intensity. In addition, FLIM can selectively filter short‐lived autofluorescence, providing enhanced contrast and sensitivity—crucial for accurate live‐cell imaging [23]. The effectiveness of FLIM critically depends on fluorophores with long lifetimes [24] that outcompete cellular autofluorescence, through time‐gated (TG) filtering [25]. Recent developments have introduced fluorophores with significantly prolonged τ_FL_, including transition‐metal complexes, lanthanides, and organic chromophores. Among the latter, triangulenium‐based fluorophores stand out for their exceptionally long lifetimes (20–25 ns), high photostability, and suitability for FLIM and TG applications [26, 27, 28, 29, 30, 31, 32, 33, 34].

As discussed above, peptidic kinase activity probes based on quenching interactions demonstrate the potential for achieving large dynamic fluorescence changes. However, to date, all reported quenching‐based peptide biosensors designed to probe kinase activity using FLIM rely on synthetic dyes with τ_FL_ in the nanosecond range, which significantly limits their sensitivity in living cells [13, 15, 16, 17]. In contrast, the implementation of long‐lifetime probes specifically engineered to fully exploit the capabilities of FLIM remains largely underexplored. In this work, we report the design of triangulenium‐based peptide biosensors for sensitive detection and quantification of kinase activity using FLIM. We focus on kinases central to T cell function, including c‐Src (Src), a non‐receptor tyrosine kinase involved in signaling and cellular activation pathways [7], and Akt1, a critical mediator of proliferation, metabolic reprogramming, and survival [35, 36]. Through a multidisciplinary approach encompassing biosensor design, photophysical characterization, *in silico* characterization, in vitro and *in extracto* assays, and *in cellulo* FLIM imaging, we demonstrate the utility of these biosensors for monitoring kinase activation in living T cells by flow cytometry. These tools represent a significant step toward the real‐time, non‐invasive analysis of kinase signaling and may ultimately contribute to identifying alterations associated with the onset of disease, monitoring changes in protein kinase activity throughout disease progression, and assessing responses to therapeutics.

## 2 Results and Discussion

### 2.1 Design of Luminescent Peptide‐Based Biosensors for Protein Kinase Activity

We have developed a new family of luminescent biosensors to probe Src and Akt1 activity. Their design entails a kinase recognition domain (the peptide sequence recognized by the kinase under study, Src or Akt1), a phosphorylatable residue (Ser or Tyr for Akt1 and Src, respectively), and a reporter fluorophore attached to a cysteine residue at the ±2 position relative to the phosphorylatable residue (Figure 1). This design is based on previously reported probes where the quenching of the fluorophore's emission is prevented upon phosphorylation, leading to an increase in fluorescence [10, 11, 12, 13, 14, 15, 16, 17]. As an advancement, we focused on introducing fluorophores with long τ_FL_ to exploit the orthogonal information provided by time‐resolved fluorescence techniques, such as FLIM. Triangulenium dyes are highly planar, aromatic carbocations that stand out for their exceptional τ_FL_ values [26, 27, 28, 29, 30, 31, 32, 33, 34]. Among the triangulenium derivatives, we focused on the commercially available, thiol‐reactive derivative of azadioxotriangulenium (ADOTA), KU530. Table 1 and Table S1 show the peptide sequences of the sensors, indicating the positions of the phosphorylatable residue and the reporter fluorophore. Once the biosensors were synthesized and purified (see Supporting Information for details), we first tested their performance in reporting phosphorylation and optimized the conditions for their use (Figure S1). We also performed a comprehensive computational study to fully elucidate the sensing mechanism.

**FIGURE 1:**
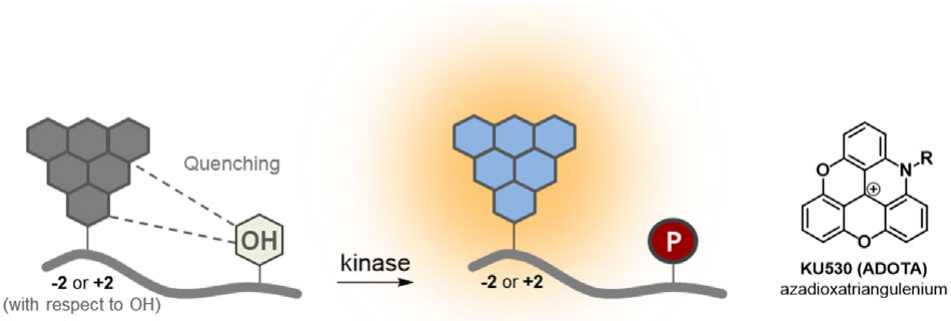
Illustration of the quenching‑based fluorescent biosensor for kinase activity containing KU530 as the reporter fluorophore. In the unphosphorylated state, the emission of KU530 is quenched; kinase‑mediated phosphorylation produces a strong fluorescence turn‑on signal.

**TABLE 1:** Fluorescence properties and fold‐intensity changes of KU530‐labeled peptide substrates. The table shows peptide sequences, fold‐intensity increases, and fluorescence‐lifetime parameters for non‐phosphorylated and phosphorylated Src and Akt1 biosensors (5 µM; PBS pH 7.4,100 mM NaCl).

Entry	Peptide sequences^a^	Fold intensity increase	τ_FL_/ns^b^
**Src‐OH**	EEC(KU530)IY*GEIEA	8.3 ± 0.2	2.64 ± 0.03
**Src‐P**	EEC(KU530)IpY*GEIEA		15.61 ± 0.04
**Akt1‐OH**	ARKRERAYS*FC(KU530)HHA	3.5 ± 0.9	6.19 ± 0.05
**Akt1‐P**	ARKRERAYpS*FC(KU530)HHA		14.62 ± 0.1

^a^ In the peptide sequences, the underlined residues are consensus substrate residues; the reporter fluorophore KU530 is placed in brackets after the corresponding Cys (C) residue used for labeling; the asterisk (*) indicates the phosphorylatable Tyr (Y) or Ser (S), and the lowercase ‘p’ indicates the phosphorylated state. The *N*‐terminus is acetylated for maintaining similarity with the actual substrate.

^b^ Amplitude‐weighted average lifetime.

### 2.2 Optimization of the Peptide Biosensors

To validate these tools and determine the dynamic range of the response, the biosensors and their corresponding fully phosphorylated versions were prepared using the KU530 triangulenium dye (see Table 1, and Supporting Information for Materials and Methods).

The potential response of the biosensors associated with the phosphorylation process was determined by comparing the fluorescence emission (intensity and fluorescence lifetime, τ_FL_) of synthetically phosphorylated variants with those of their corresponding unphosphorylated counterparts. We refer to the original sensors as **Akt1‐OH** and **Src‐OH**, where the name reflects both the target kinase and the ─OH group available for phosphorylation. The phosphorylated forms are denoted **Akt1‐P** and **Src‐P**, indicating that the ─OH group has been replaced by a phosphate group.

Additionally, we evaluated the performance of the biosensors using both cuvette‐based spectrofluorometry and plate reader measurements. These complementary approaches allowed us to confirm their reproducibility and sensitivity under different experimental conditions, demonstrating the robustness of the probes for quantitative analysis. The developed biosensors showed a significant fluorescence increase in the phosphorylated variants: 8.3 ± 0.2‐fold for the Src biosensor and 3.5 ± 0.9‐fold for the Akt1 biosensor (Table 1 and Figure 2), thus validating their potential utility for enzymatic assays due to their enhanced response. Furthermore, they exhibited remarkable increases in their τ_FL_ values, increasing from 2.64 to 15.61 ns for Src, and from 6.19 to 14.62 ns for Akt1, for the ‐OH control and phosphorylated form, respectively (Table 1). This indicated that our biosensors could be used to probe kinase activity via FLIM with high sensitivity and a superior signal‐to‐noise ratio, as the τ_FL_ values are significantly longer than those of cellular autofluorescence [20, 25].

**FIGURE 2:**
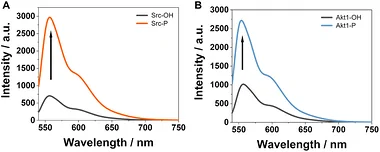
Representative fluorescence emission spectra of the biosensors and their fully phosphorylated controls. (A) **Src‐OH** (black) and **Src‐P** (orange); (B) **Akt1‐OH** (black) and **Akt1‐P** (blue). All spectra (*λ*_ex_ = 530 nm) were collected from solutions of the peptides at 5 µM, diluted in PBS pH 7.4 NaCl 100 mM.

### 2.3 Computational Study of the Sensing Mechanism

Early reports of similar peptide‐based kinase probes did not fully elucidate the photophysical mechanism underlying the sensing response. An early example of Tyr‐kinase probe demonstrated the involvement of π‐stacking interactions in the quenching of the fluorophore's emission by the Tyr residue, with subsequent recovery of luminescence upon phosphorylation [10]. Other examples attributed variations in the fluorescent tag upon phosphorylation to changes in the microenvironment [15]. However, in many previous studies, the underlying mechanism of the recovery of luminescence upon phosphorylation was not addressed from a photophysical perspective [13, 16, 17].

In contrast, we aimed to elucidate the working principle of our probes, focusing on the photophysics of the triangulenium reporter. In fact, previous works on triangulenium fluorophores tagging proteins reported partial quenching of the luminescence that was assigned to potential photoinduced electron transfer (PET) with aromatic amino acids, such as tryptophan [37, 38]. However, for other fluorophores, it has been found that this effect can occur even when the amino acid lacks aromatic rings, as the electronic interactions between the electron donor and acceptor are not limited to π–π stacking; the HOMO and LUMO orbitals participating in the process also play a fundamental role [39]. Therefore, to assist in interpreting our experimental findings and elucidate the sensing mechanism, we conducted an extensive computational study involving the peptide biosensors and their phosphorylated states.

First, we performed a structure search on the peptides **Akt1‐OH** and **Src‐OH**, along with their phosphorylated counterparts (**Akt1‐P** and **Src‐P**). This process involved an initial conformational search in water using the GFN‐FF force field implemented in CREST software (see Computational Details in SI). Tables S3–S6 show the relative energies of the conformers found below 6 kcal/mol. Given the many degrees of freedom leading to multiple local minima, a total of 28 conformations were found for the **Akt1‐OH** peptide and 11 conformations for the phosphorylated peptide (**Akt1‐P**). For the Src peptide, 128 and 314 conformations were found for the non‐phosphorylated (**Src‐OH**) and phosphorylated (**Src‐P**) states, respectively. These ensembles of structures provide information on the conformational flexibility of the peptide chain. Subsequent optimization of the geometry for the lowest‐energy conformer was performed using Density Functional Theory (DFT) at the ωB97xD/6‐31G* level, a long‐range corrected functional. We then performed time‐dependent DFT (TD‐DFT) to estimate the associated transitions upon absorption of light (Table S2). In the stable conformations of all four peptides, the lowest unoccupied molecular orbital (LUMO) is always localized over the triangulenium fluorophore, and the excitation transitions arise from inner energy orbitals that are also localized at the triangulenium moiety (Figures S2–S5).

Regarding the quenching mechanism behind both sensors, we propose the existence of a PET process between surrounding electron donor fragments and the triangulenium moiety. When analyzing the plausible sources of PET process, it is important to take into consideration not only the existence of potential electron donor groups in the spatial surroundings of the acceptor fragment but also that orbital overlapping between donor and acceptor molecular fragments is a mandatory condition that must be fulfilled for the photon electron transfer to occur [40]. Thus, visual inspection of the molecular orbitals involved not only in the transitions but also in the energy levels close to the ground/parting state allow us to corroborate both spatial proximity and overlapping capabilities.

For the case of **Src‐OH**, the close inspection of the optimized peptide structure revealed a clear π‐stacking interaction between the KU530 moiety and the phenol group of the Tyr amino acid (Figure 3A). The distance between the two aromatic moieties is in the range of <4 Å, typical values for π–π interactions. Importantly, phosphorylation of the Tyr hydroxyl group drastically moves the Tyr moiety away from the ADOTA fluorophore in the peptide **Src‐P**. To consider the influence of conformational dynamics of the peptidic chains, we analyzed the ensemble of structures obtained using the CREST software (Tables S5 and S6) focusing on the inter‐moiety distance. Figure 3B shows that the distribution of distances between the KU530 and the Tyr aromatic moieties lays mostly in the range of π‐stacking interactions for **Src‐OH**, whereas the Tyr is shifted to distances >8 Å for the phosphorylated peptide. This process is remarkably similar to previous reports of tyrosine‐kinase probes [10, 41]. Importantly, the decreased τ_FL_ value of the **Src‐OH** probe supports the dynamic nature of the quenching, so that the π‐stacking is the requirement to place the Tyr phenol group in close contact to KU530 prior to a PET process. In fact, this stacking interaction is accompanied by an appreciable molecular orbital overlap between HOMO–3, involved in the absorption transition at the triangulene (Figure S2), and the two subsequent higher‐energy orbitals (HOMO–2 and HOMO–1), in which the Tyr residue is directly involved. Consequently, given this spatial proximity, the energy difference (∼0.2 eV), and concomitant orbital overlap, a substantial electron transfer can be expected. Nevertheless, upon phosphorylation this stacking interaction gets hindered due to conformational changes developed within the structure to accommodate the negative charged phosphate group near the positive charge triangulenium. Thus, as the phosphorylated tyrosine group gets pulled over the periphery of the peptide core, orbital overlapping is highly diminished, and PET process gets almost fully neglected. Therefore, we assign the main working mechanism of the **Src‐OH** probe to a quenched state due to PET from the Tyr, facilitated by π‐stacking interactions, that are hindered upon phosphorylation.

**FIGURE 3:**
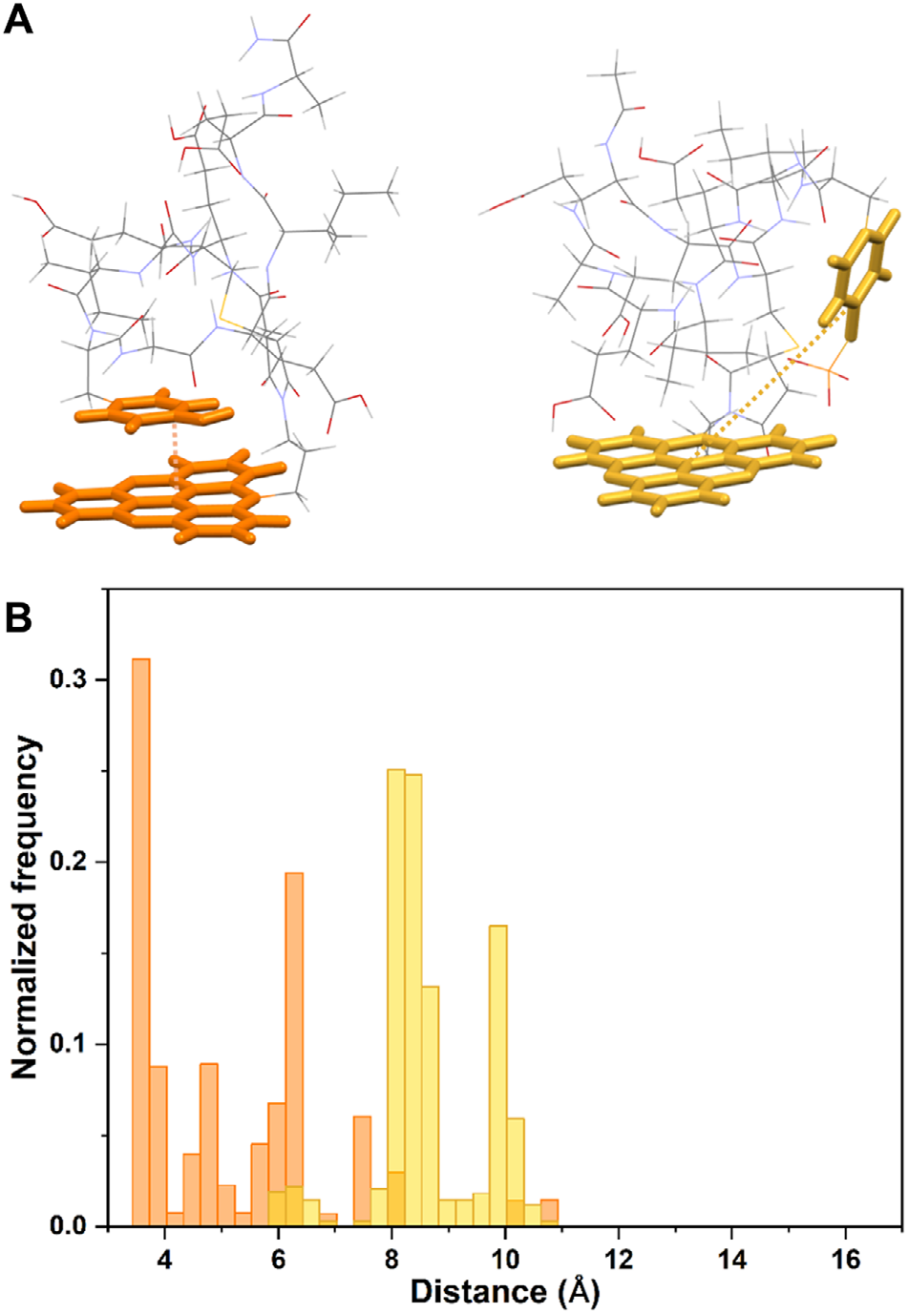
A) CREST‐extracted structure and subsequently optimized structure, calculated at the ωB97xD/6‐31G* level of theory, of **Src‐OH** and **Src‐P**. The KU530 and the aromatic side group of Tyr are highlighted. B) Distance distribution, weighted by the Boltzmann function, between the KU530 and the aromatic side group of Tyr in **Src‐OH** (orange) and **Src‐P** (yellow).

In contrast, the sequence of the **Akt1‐OH** peptide contains different side groups that can cause PET quenching. We first focused on the Tyr residue near the phosphorylatable Ser. The analysis of the CREST stable conformations showed a major population at a distance ∼8.5 Å that gets broader and delocalized upon phosphorylation (Figure S6). Although these distances may be sufficient for a slow PET to occur, it is less likely that the Tyr is the major source of quenching. Therefore, we investigated other potential sources of quenching based on PET. Interestingly, decreased quenching at acidic pH for **Akt1‐OH** suggest involvement in PET from the two nearby His, imidazole side groups (Figure S8). The analysis of the molecular orbitals of **Akt1‐OH** revealed at least two molecular orbitals, HOMO–5 and HOMO–6, that share a similar distribution over the triangulenium moiety and the His‐His motif (Figure 4A). We propose that this dual His motif may act as the primary electron donor group, not necessarily due to the intrinsic electron transfer capability of isolated His [42], but rather because the His‐His pair can function as an enhanced PET relay. This cooperative effect stems from its capacity to stabilize partial positive charges via aromatic delocalization, or even to act as an inducer of proton‐coupled electron transfer (PCET) [43, 44]. In contrast, in the phosphorylated **Akt1‐P** peptide, the imidazole rings of the two His residues are located closer to the phosphate group, which stabilizes their electron density and contributes to a delocalized molecular orbital that lacks any participation from the triangulenium fragment (Figure 4B). Importantly, the HOMO–22, from which the main absorption transition originates (Table S2), does not exhibit electron coupling with the His residues. Furthermore, upon phosphorylation of the Ser residue, the two His residues become spatially separated from each other (Figure 4B and Figure S7), which prevents the charge stabilization of the His‐His motif. This increased inter‐His distance, combined with the notable lack of orbital contribution from the triangulenium moiety, likely hinders the orbital overlap between states, thereby drastically diminishing the PET efficiency.

**FIGURE 4:**
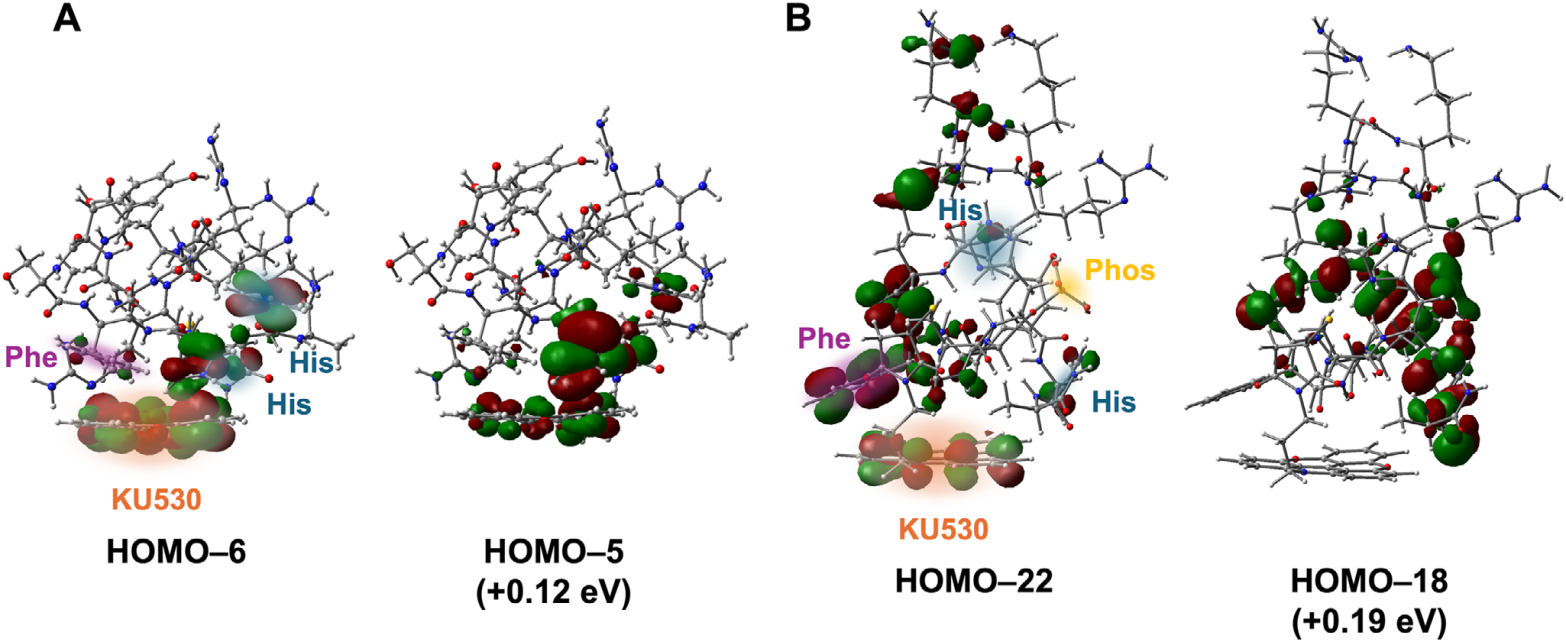
Molecular orbital representation (isocontour plots 0.02 a.u.) and optimized structure of **Akt1‐OH** and **Akt1‐P**, calculated at the ωB97xD/6‐31G* level of theory. A) For **Akt1‐OH**, the figure depicts the HOMO‐6 of the triangulenium from where the main absorption transition starts (Table S2) and the HOMO‐5 containing coupled electron density at the His residue responsible of PET quenching (see Figure S4 for other molecular orbitals and the energy diagram). B) For **Akt1‐P**, the figure depicts the HOMO‐22 from where the absorption transition originates (Table S2) and the HOMO‐18 containing notable electron density at the His residue and the phosphate group (see Figure S5 for other molecular orbitals and the energy diagram). In all cases, the KU530, the aromatic Phe residue, the two His residues, and the phosphate group (Phos) are highlighted.

### 2.4 In Vitro Validation of the Peptide Biosensors

The enzymatic performance of the developed Akt1 and Src peptide biosensors in phosphorylation assays was evaluated in vitro in the presence of active recombinant enzymes and the required ATP and Mg^2+^ (Figure S1). The kinetic response of phosphorylation was determined by monitoring fluorescence intensity changes as a function of substrate concentration, and the data were fitted to the Michaelis–Menten equation (Figure 5). Both biosensors exhibited typical saturation kinetics, confirming their suitability as kinase substrates (Figure S9). For Akt1, we used 50 ng of kinase and an **Akt1‐OH** biosensor concentration of 0.25–7.5 µM; the apparent Michaelis constant (*K*_M_) was 0.4 ± 0.1 µM, indicating high affinity for the biosensor, while the maximum catalytic rate (*V*_max_) reached 2.8 ± 0.2 min^−1^, consistent with efficient phosphorylation under physiological conditions. Similarly, using 100 ng of kinase and a **Src‐OH** biosensor concentration of 0.25–20 µM, the Src biosensor displayed a *K*_M_ of 2.8 ± 0.8 µM and *V*_max_ of 45.6 ± 4.5 min^−1^. Remarkably, their binding affinities exceed those reported for the corresponding parent peptides [45, 46], demonstrating that the incorporation of the reporter triangulenium KU530 does not compromise substrate recognition, thus supporting their application for quantitative kinase activity measurements.

**FIGURE 5:**
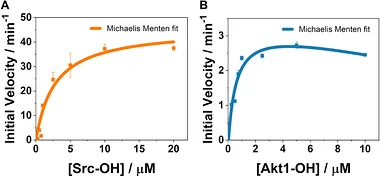
Michaelis–Menten kinetic analysis of the **Src‐OH** (A) and **Akt1‐OH** (B) biosensors. The initial velocity (min^−1^) values were obtained through the analysis of the kinetics traces in Figure S9. Lines represent nonlinear fittings to the Michaelis–Menten equation. Error bars represent s.d. from 3 independent repetitions.

### 2.5 In Vitro Probing of Endogenous Kinase Activity in HeLa Cell Extracts

Next, the biosensors were evaluated in a complex environment using cell extracts as a source of endogenous kinases. Endpoint kinase activity assays were performed in HeLa cell extracts, cytoplasmic or membrane fractions (see Supporting Information), to assess the performance of the biosensors in biological media, providing an essential intermediate step between measurements with recombinant kinases and *in cellulo* studies. Owing to the complex biochemical composition of extracts –which preserve elements of native regulation, cofactor availability, and protein–protein interactions– they provide a more physiologically relevant environment while retaining experimental tractability. However, this complexity also introduces challenges, as extracts contain undefined mixtures of kinases, phosphatases, proteases, and other biomolecules that can attenuate or complicate substrate‐based fluorescence readouts.

Screening experiments revealed a clear concentration dependence, with 10 µg of extract providing the highest phosphorylation‐dependent signal enhancement (1.4‐fold and 1.3‐fold for the Src and Akt1 biosensors, respectively; Figure S10). To establish specificity, extracts were preincubated with selective kinase inhibitors, LY294002 (1–20 µM) as Akt1 inhibitor and Dasatinib (25–200 nM) to inhibit Src activity (Figure S11) [47, 48]. Our results showed that the inhibitors suppressed biosensor phosphorylation at nearly all concentrations, leading to fluorescence levels comparable to the negative controls. The most pronounced effects were observed with 10 µM LY for Akt1 and 50 nM Das for Src (Figure 6). These assays confirmed that the fluorescence changes originate from the genuine modulation of kinase activity and that the inhibitors retain efficacy within the biochemical milieu of the extract.

**FIGURE 6:**
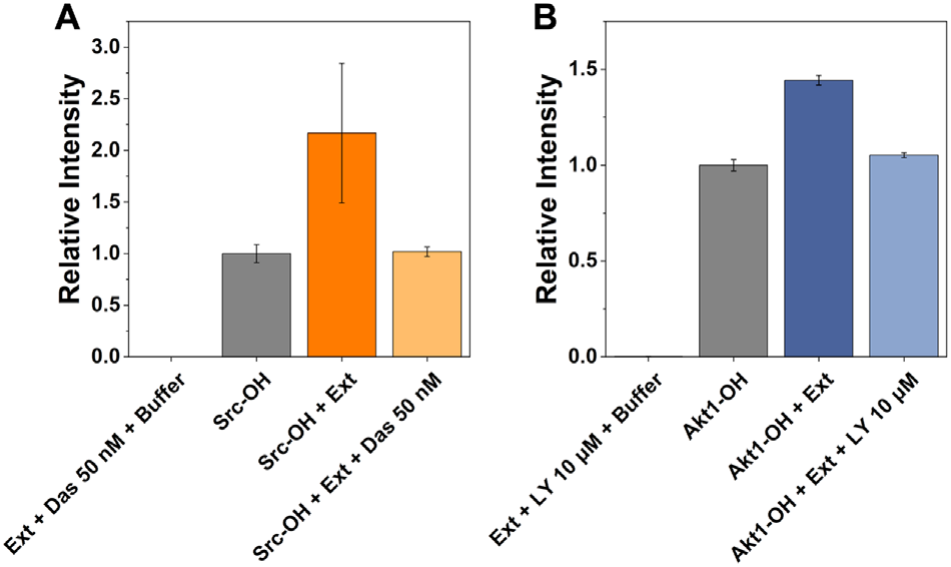
Endpoint extract assays for endogenous kinase activity of Src (A) and Akt1 (B) were performed using the peptide biosensors (5 µM). Emission intensity values were normalized to the unphosphorylated biosensors in buffer (**Src‐OH** or **Akt1‐OH**, grey bars). The emission intensity of the probes increased in the presence of 10 µg standard cell extracts (dark‐colored bars; **Src‐OH**, dark orange; and **Akt1‐OH**, dark blue). The signal decreased with increasing concentrations of their respective inhibitors, 50 nM Das or 10 µM LY, respectively (light‐colored bars), reaching the same levels as the unphosphorylated controls. Fluorescence emission at 560 nm (*λ*_ex _ =  530 nm). Error bars represent s.d. from 3 independent experiments.

### 2.6 *In Cellulo* Validation by FLIM Microscopy

The performance of the biosensors *in extracto* robustly supports their use for *in cellulo* applications. Moreover, the long τ_FL_ values of the triangulenium dye KU530 make them particularly suitable for cellular bioimaging using FLIM [28, 31, 32, 33, 34]. We applied advanced multidimensional FLIM to assess the biosensors’ ability to report physiologically relevant kinase signaling within living cells. Importantly, to ensure efficient cellular internalization, the peptides were covalently modified by adding Pep1, a well‐known cell‐penetrating peptide, to the *N*‐terminus [49, 50]. First, we checked that the modified peptide biosensors **Src‐Pep1‐OH** and **Akt1‐Pep1‐OH** (Table S1) retained activity, responding to endogenous kinases in cell extracts (Figure S12).

To facilitate homogeneous cellular uptake of the various peptides, the modified peptides were encapsulated within Pep1‐micelles, serving as the primary drivers of internalization [49, 50] (see Methods in Supporting Information). In the microscopy assays, the response of the biosensors was compared with non‐phosphorylatable control peptides (**Src‐Pep1‐Ala** and **Akt1‐Pep1‐Ala**), in which the phosphorylatable residue was substituted with Ala.

We also performed cytotoxicity assays (Figure S13) that showed no measurable toxicity up to 25 µM of biosensor. Real‐time fluorescence measurements revealed rapid intracellular accumulation following first‐order kinetics, reaching maximal fluorescence within 30 min (Figure S14). These preliminary assays validated both the biocompatibility and efficient internalization of the biosensors, providing the necessary foundation to proceed with functional activity measurements.

In our FLIM experiments using live HeLa cells, we focused on τ_FL_ values and the TG‐filtered fluorescence intensity (*I^TG^*), where the initial 5 ns after the excitation pulse were removed to eliminate cellular autofluorescence. The **Src‐Pep1‐OH** biosensor exhibited both higher *I* ^TG^ and longer τ_FL_ values compared to its Ala containing counterpart (Figure 7 and Figure S15). Specifically, the biosensor displayed a 1.6‐fold increase in *I* ^TG^ and a notable lifetime extension from 7.2 ± 0.7 ns (**Src‐Pep1‐Ala**) to 8.8 ± 0.6 ns (**Src‐Pep1‐OH**) (Figure 7). These increases confirmed intracellular phosphorylation of the biosensor by the endogenous target kinase. The lifetime values are averaged across all collected images and from the regions of interest containing cells as a whole. However, close examination of the images (Figure 7 and Figure S15) revealed regions exhibiting particularly long lifetimes. This suggests the presence of varying levels of phosphorylation of the peptides and subsequent subcellular localization. This heterogeneity accounts for the reduced differences in the average lifetime values compared to those obtained in vitro. Pre‐incubation with the Src inhibitor Das (40 nM) significantly reduced both intensity and τ_FL_, reaching levels comparable to those of the negative control, thus confirming the specificity of the Src biosensor for its target kinase.

**FIGURE 7:**
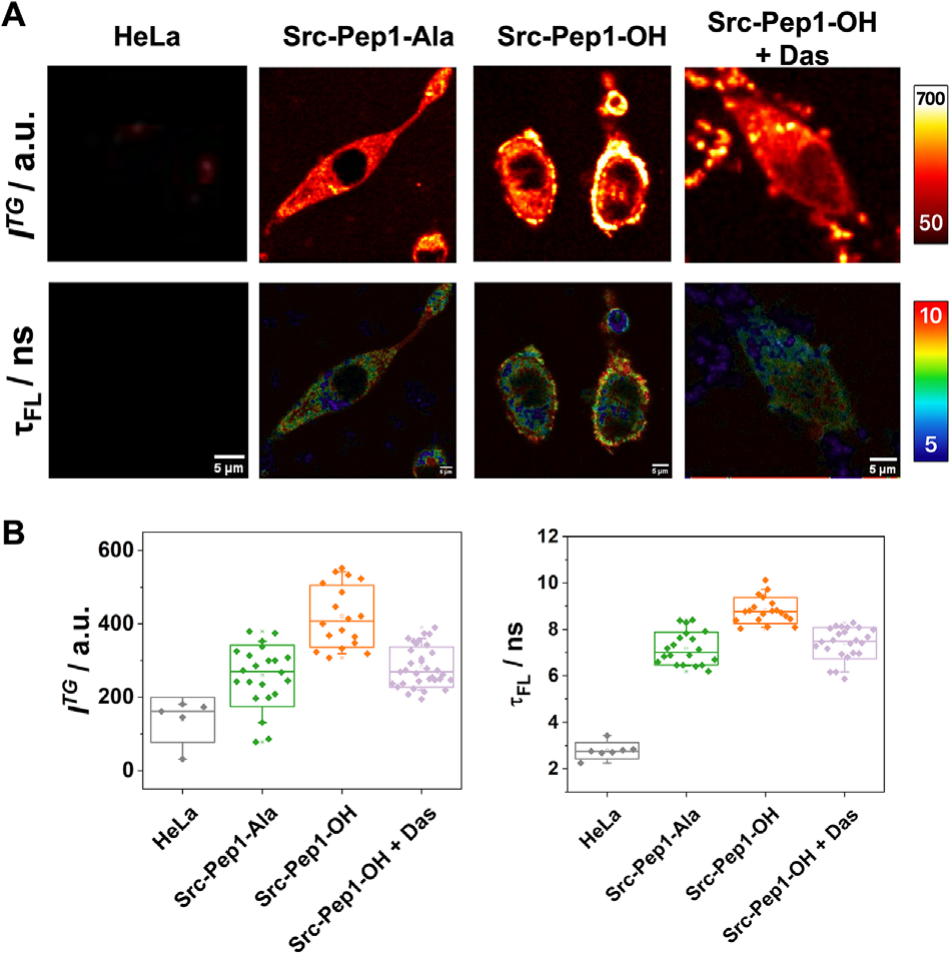
(A) TG intensity (*I* ^TG^, top) and τ_FL_ (bottom) imaging of the Src activity biosensor in HeLa cells: (i) untreated HeLa cells; (ii) non‐phosphorytalable **Src‑Pep1‑Ala** control; (iii) **Src‑Pep1‑OH** biosensor; (iv) **Src‑Pep1‑OH** biosensor in the presence of 40 nM Das. Scale bars represent 5 µm. (B) Box plots of *I* ^TG^ (left) and τ_FL_ values (right) from different images for each condition. *I* ^TG^ was defined in a detection time widow of 5 ns after the excitation pulse. Boxes represent s.d. while whiskers represent 90% of the population from all the images collected from at least 2 independent repetitions.

Similarly, FLIM confirmed that the phosphorylatable peptide **Akt1‐Pep1‐OH** exhibited higher TG intensity and τ_FL_ values than the **Akt1‐Pep1‐Ala** control (Figure 8 and Figure S16), indicating intracellular phosphorylation by the endogenous kinase. Quantitatively, we observed a 2.7‐fold increase in fluorescence intensity and a τ_FL_ increase from 9.2 ± 2.9 to 13.2 ± 3.4 ns. As previously explained, these lifetimes are averaged across all cells. However, visual inspection of the images enables the detection of subcellular heterogeneity and regions of interest with significantly higher lifetime values, which correlate with phosphorylation activity. Treatment with the Akt1 activator insulin (50 nM) [51, 52] resulted in significantly higher luminescence intensity and τ_FL_, while the inhibitor LY294002 (LY, 10 µM) caused a marked decrease, yielding values similar to those of the negative control.

**FIGURE 8:**
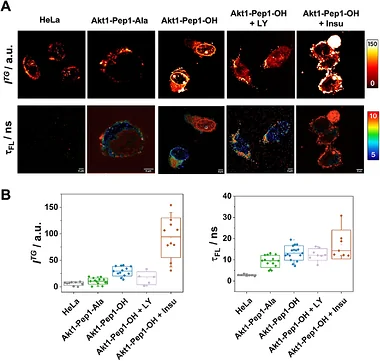
(A) TG intensity (*I* ^TG^, top) and τ_FL_ (bottom) imaging of the Akt1 activity biosensor in HeLa cells: (i) untreated HeLa cells; (ii) non‐phosphorytalable **Akt1‑Pep1‑Ala** control; (iii) **Akt1‑Pep1‑OH** biosensor; (iv) **Akt1‑Pep1‑OH** biosensor in the presence of 10 µM LY; (v) **Akt1‑Pep1‑OH** biosensor in the presence of 50 nM insulin (Insu). Scale bars represent 5 µm. (B) Box plots of *I* ^TG^ (left) and τ_FL_ values (right) from different images for each condition. *I* ^TG^ was defined in a detection time widow of 5 ns after the excitation pulse. Boxes represent s.d. while whiskers represent 90% of the population from all the images collected from, at least 2 independent repetitions.

### 2.7 Akt1 Kinase Activity in Primary T Cells

To demonstrate the application of the platform beyond a proof‐of‐concept, we employed the Akt1 biosensor to probe endogenous kinase activity in primary T cells. Akt1 was selected because it represents a central node in T cell biology, integrating T cell receptor (TCR) and co‐stimulatory signals with metabolic reprogramming, survival, proliferation, and effector differentiation. Upon TCR/CD28 engagement, PI3K activation drives the accumulation of PIP_3_ and the subsequent recruitment of Akt1 to the plasma membrane. Once localized, Akt1 is phosphorylated at residues T308 and S473, enabling downstream signaling through mTORC1, inhibition of GSK3β, and the regulation of transcriptional programs that control cell growth and cytokine production [53, 54]. Consequently, Akt1 serves as a critical regulator of the metabolic switch that supports clonal expansion and the acquisition of effector functions. Furthermore, dysregulated Akt signaling is implicated in autoimmunity, chronic infection, and cancer, underscoring the clinical importance of monitoring its dynamics in living immune cells [55].

We initially confirmed the internalization of the **Akt1‐Pep1‐OH** biosensor in isolated mouse T cells using live‐cell confocal microscopy. Figure 9 demonstrates clear intracellular localization within 30 min of incubation (steps 1–2). Given the pivotal role of Akt1 in coupling antigen recognition to metabolic activation, cytokine production, and clonal expansion, we monitored fluorescence in real time before and after stimulation with soluble αCD3 and αCD28 antibodies. TCR engagement via αCD3 initiates proximal signaling cascades, while αCD28 provides the necessary co‐stimulatory input to fully activate PI3K and downstream Akt1 signaling. Under these conditions, Akt1 phosphorylation increases rapidly, driving glycolytic reprogramming and transcriptional programs required for proliferation and effector differentiation [56]. Following the attainment of uptake equilibrium (∼30 min; Figure 9, step 2), stimulation elicited a >2‐fold increase in fluorescence intensity over the subsequent 20 min (Figure 9, step 3), consistent with phosphorylation‐dependent sensor activation.

**FIGURE 9:**
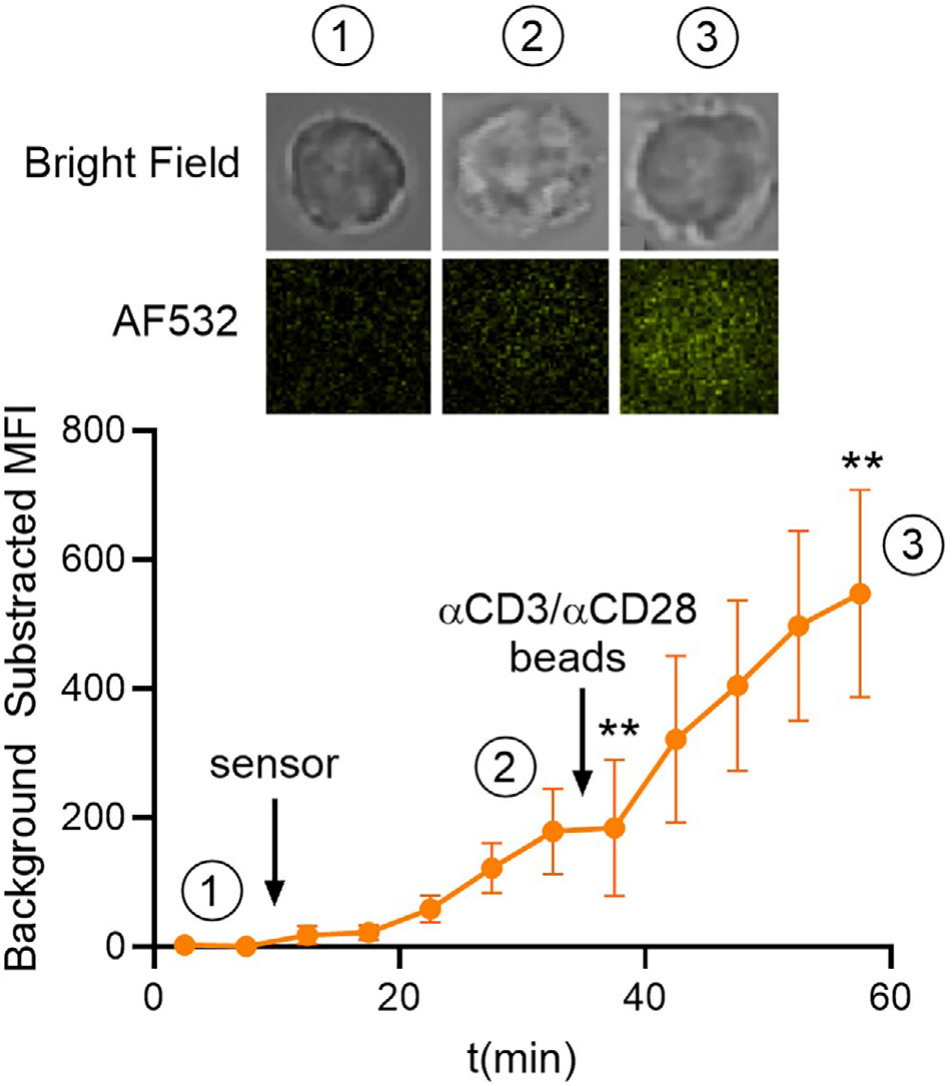
Live‐cell confocal microscopy images of isolated mouse T cells. The images illustrate the intracellular localization of the **Akt1‐Pep1‐OH** sensor shortly after exposure (step 1–2) and the subsequent Akt1 activation induced by αCD3/αCD28 stimulation (step 2–3). The background‐subtracted mean fluorescence intensity (MFI) is plotted, with error bars representing the s.d. of 3 independent measurements. ** indicates significant differences (*p* < 0.01) using an ANOVA test.

Notably, the signal was cumulative, effectively amplifying transient kinase activity into a stable, detectable readout. This feature is particularly advantageous for capturing rapid signaling events that might otherwise remain undetected, especially when Akt1 activation is transient or subtle. Because the signal accumulates over time, it also facilitates the retrospective identification and sorting of cell populations that have experienced prior Akt1 activation

To determine whether Akt1 activity could be quantified simultaneously across distinct T cell subsets, we incubated mouse splenocytes with the **Akt‐Pep1‐OH** biosensor and stained for CD4 and CD8 surface markers. Flow cytometry revealed an approximately 5‐fold increase in Akt1 signal in both CD4^+^ and CD8^+^ T cells after 30 min compared to the control (Figure 10A). We then examined stimulus‐specific differences. After a 30‐min loading phase, cells were washed and stimulated either through TCR engagement (αCD3/αCD28) or via direct activation of downstream protein kinase C (PKC) using the diacylglycerol analog phorbol 12‐myristate 13‐acetate (PMA). As expected, αCD3/αCD28 stimulation robustly increased Akt1 activity in both CD4^+^ and CD8^+^ subsets (15%–40% above baseline) (Figure 10). PMA also induced a measurable Akt1 increase in CD4^+^ cells, albeit to a lesser extent than TCR stimulation, which is consistent with the bypass of proximal receptor‐mediated PI3K recruitment. In contrast, PMA did not elicit a detectable Akt1 signal in CD8^+^ cells (Figure 10B). While the precise mechanism underlying this divergence remains to be fully elucidated, it likely reflects intrinsic differences in signaling thresholds or pathway organization between CD4^+^ and CD8^+^ subsets. Collectively, these results demonstrate that the biosensor can resolve subset‐specific and stimulus‐dependent differences in endogenous Akt1 activity in primary T cells, providing a robust and quantitative tool to interrogate signaling heterogeneity at single‐cell resolution.

**FIGURE 10:**
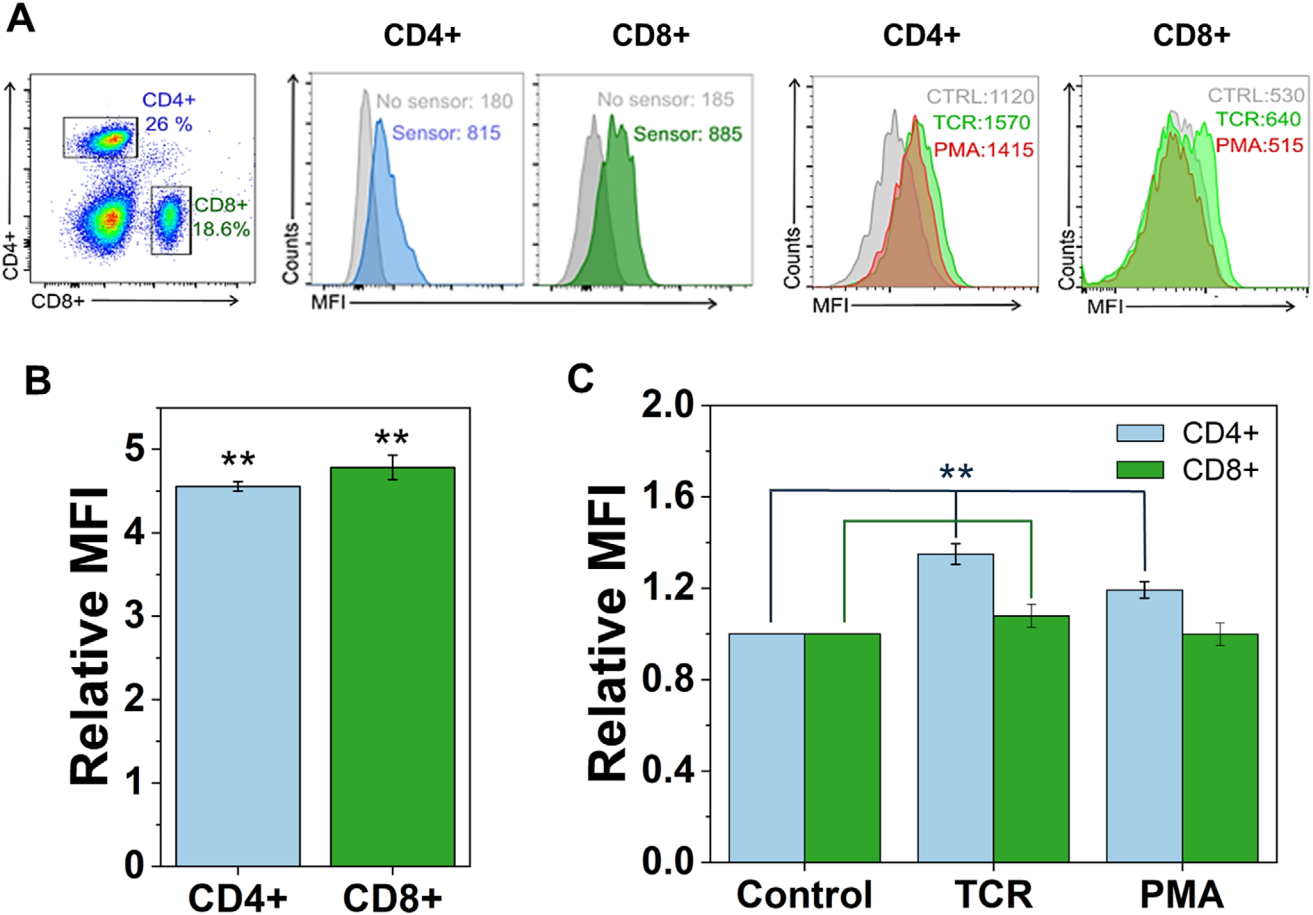
Application of the **Akt1‐Pep1‐OH** sensor for monitoring Akt1 activity in primary T cells. (A) Representative scatter plot showing gating of CD4^+^ and CD8^+^ T cell populations, and overlaid histograms of Akt1 sensor fluorescence (MFI in the FITC channel) after 30 min of incubation with sensor or control (background without sensor). Pair 1: For CD4^+^ T cells: control, gray; sensor, blue. For CD8^+^ T cells: control, gray; sensor, green; Pair 2: MFI distributions for control (gray, prior activation) and stimulated cells (αCD3/αCD28, green; PMA, red). (B) Flow cytometry analysis of sensor uptake in splenocytes. Bar graph summarizing the mean fold‐change in fluorescence intensity in both subsets, compared to no sensor. (C) Flow cytometry analysis of Akt1 activation in CD4^+^ (blue) and CD8^+^ (green) T cells, showing MFI distributions for control (prior activation) and stimulated cells (αCD3/αCD28, PMA), with the corresponding bar graph summarizing fold‐changes relative to the values of non‐stimulated conditions. Statistical significance was determined by ANOVA; **p < 0.01.

## 3 Discussion

Over the past decade, a central challenge in biology has been to monitor protein kinases and their activity in their native cellular environment. In this context, fluorescent peptide‐based biosensors are powerful tools for probing protein kinase activity, particularly those that exploit quenching by phosphorylatable Tyr residues to generate large luminescence turn‐on responses [10, 11, 12, 13, 14, 15, 16, 17, 57, 58]. For instance, the group of David Lawrence developed a pyrene‐based biosensor to probe the activity of the Tyr kinase Src in vitro [10]. Phosphorylation of Tyr disrupts the dye‐Tyr stacking interaction, thereby leading to a 4.7‐fold enhancement in the fluorescence emission intensity. However, the short excitation and emission wavelengths of pyrene (*λ*_ex_ = 340 nm, *λ*_em_ = 380 nm) limited its applicability in cellular assays due to interference from cellular autofluorescence. Consequently, this strategy was further extended to other fluorophores with longer excitation and emission wavelengths, such as Cascade Yellow (*λ*_ex_ = 400 nm, *λ*_em_ = 550 nm), which displayed a 2.0‐fold fluorescence increase upon phosphorylation. Upon microinjection, the corresponding Tyr‐“caged” sensor (*λ*_ex_ = 400 nm, *λ*_em_ = 550 nm) enabled monitoring of Src activity *in cellulo* with good temporal control over the sensing activity [57]. In a similar approach, a pair of Abl and Lyn sensors, incorporating Cascade Yellow (*λ*_ex_ = 400 nm, *λ*_em_ = 550 nm) and Oxazine (*λ*_ex_ = 667 nm, *λ*_em_ = 684 nm), respectively, were developed for the simultaneous visualization of the corresponding kinases in imatinib‐resistant and ‐sensitive cell lines [12]. Nevertheless, all these systems rely primarily on photoluminescence intensity measurements which, while highly sensitive, are inherently susceptible to systematic errors from background fluorescence in biological media, ultimately limiting their quantitative robustness and reliability in *in cellulo* settings.

In this context, FLIM microscopy emerges as a powerful approach for intracellular quantification, as it enables the simultaneous measurement of fluorescence intensity and τ_FL_ [20, 21, 22]. Moreover, TG approaches can efficiently filter short‐lived autofluorescence, improving contrast and sensitivity [25], which is crucial for accurate live‐cell imaging. However, the effectiveness of FLIM is dependent on luminescent probes with long τ_FL_ values [24] to effectively surpass cellular autofluorescence. Unfortunately, only a limited number of FLIM‐compatible quenching‐based peptide biosensors have been reported. For instance, Damayanti et al. described two quenching‐based peptide biosensors to monitor VEGFR2 and FAK phosphorylation activities in real time. Nevertheless, their short fluorescence lifetimes (<5 ns) inherently restricted their sensitivity and resolution in cellular environments [15, 16].

To overcome this limitation and extend the applicability of kinase biosensors to FLIM imaging, we have presented herein a new family of quenching‐based peptide biosensors incorporating long‐τ_FL_ triangulenium dyes to probe protein kinase activity through sensitive changes in their luminescence (intensity and τ_FL_). Specifically, the commercially available triangulenium derivative KU530, characterized by its strong yellow emission (*λ*_ex_ = 530 nm, *λ*_em_ = 560 nm), extended τ_FL_ (20–25 ns), and robust photostability was employed as the reporter fluorophore, enabling robust discrimination between phosphorylated and substrate peptides through TG intensity and τ_FL_. In this work, we selected target kinases —Akt1 and Src— that play key roles in regulating T cell functions and are well‐established cancer biomarkers. Although the response mechanism of our probe for Src, a Tyr‐kinase, primarily relies on quenching driven by Tyr, similar to previous probe designs [10, 11, 12, 13, 14, 15, 16, 17, 57, 58], an additional advantage of using triangulenium dyes as reporter fluorophores is their redox properties, which make them susceptible to additional PET quenching. This has enabled us to design a probe for the activity of Akt1 kinase, a Ser kinase. We have thoroughly investigated the sources of quenching and the mechanism for luminescence recovery. Our findings indicate that π‐stacking interactions facilitate PET‐driven quenching from the Tyr residue in the Src probe, whereas PET from the His‐His motif is the primary source of quenching for the Akt1 probe. The modular and versatile nature of these probes expands the design possibilities, making them broadly applicable to nearly any Ser or Tyr kinase of interest. Importantly, these probes display a strong luminescent response upon kinase phosphorylation while maintaining high affinity for their target kinases. Remarkably, their binding affinities exceed those reported for the corresponding parent peptides, indicating that the incorporation of the KU530 dye does not impair substrate recognition by the kinase [45, 46]. This adaptability enables straightforward customization of the sensing platform while preserving its core signaling features, facilitating the development of tailored biosensors for diverse biological applications.

Furthermore, these triangulenium‐based biosensors are not limited to advanced FLIM microscopy; they are inherently versatile, delivering robust performance at multiple imaging and detection platforms for monitoring kinase activity across multiple levels of biological complexity, from in vitro assays with recombinant enzymes to physiologically relevant measurements in cell extracts containing endogenous kinases. Indeed, in *in extracto* assays, the new biosensors maintain robust performance despite endogenous autofluorescence and the complexity of enzymatic environments. Their high affinity and sensitivity, as well as their broad dynamic range, make them ideally suited for detecting kinase activity changes that might be overlooked by conventional protein expression analyses, and for applications in high‐throughput screening assays. These sensors also enable the reliable monitoring of kinase inhibition by small molecules, providing an effective approach to evaluate inhibitor efficacy under physiologically relevant conditions.

The probes provided robust and reliable fluorescence intensity–based readouts using conventional confocal microscopy, flow cytometry, and plate‑based assays. This dual compatibility is particularly valuable, as it ensures that the biosensors can be deployed in research settings with varied instrumentation, ranging from advanced microscopes to standard fluorescence setups. Thus, rather than restricting the technology, the long‑lifetime triangulenium scaffold expands the operational window of the biosensors, enabling FLIM when available while maintaining strong signal‐to‐background ratios in more common equipment—an important practical advantage for broad adoption across different laboratories.

Remarkably, our biosensors operate effectively under physiological ATP and Mg^2^^+^ levels, providing a valuable platform for examining kinase‐mediated regulation in T cells. Specifically, our Akt1 biosensor was successfully applied to monitor protein kinase activity in T cells by flow cytometry, demonstrating high specificity and sensitivity in capturing basal Akt1 activity, detecting dynamic changes upon kinase activation, and distinguishing differences between CD4^+^ and CD8^+^ T cell populations. Therefore, these tools hold significant potential for identifying specific subsets of T cells, which could have an impact on improving T cell‐based therapies. These results demonstrate that our sensors open new avenues in biomedical fields, such as immunology. We anticipate broader applications in biomedical research, including cancer, where they may enable the early detection of alterations in cell proliferation and facilitate the design of smart, personalized diagnostics for monitoring disease progression and therapeutic responses.

## 4 Conclusions

Monitoring protein kinases and their activity in their native cellular environment, which enables continuous tracking of their spatiotemporal dynamics, provides insights into their regulatory roles in signaling pathways. While traditional discontinuous and invasive methods based on the extraction and purification of proteins from cell lysates, as well as strategies using antibodies, proteomics, or radioactivity, allow the detection of specific protein kinases, these approaches do not provide any information about their dynamic nature. The development of sensitive and tailored tools based on luminescent sensors, together with the latest advances in bioimaging technologies, offers the opportunity to monitor the dynamic processes of protein kinases in living cells in a sensitive and non‐invasive manner, with high spatial and temporal resolution.

Taken together, our results establish a family of peptide biosensors, based on triangulenium reporters, as a powerful tool for quantitative kinase activity measurements in complex extracts and live cells. Its compatibility with both advanced FLIM microscopy and flow cytometry enables spatially resolved single‐cell imaging as well as high‐throughput population analysis, offering a practical alternative to genetically encoded FRET sensors and protein‐based affinity reporters. Likewise, their capacity for the analysis of kinase activity across immune subpopulations underscores the potential of the sensors for detailed immune profiling.

By uniting long fluorescence lifetimes with modular substrate recognition, this platform offers a broadly adaptable and scalable strategy for probing kinase biology and opens multiple avenues for translational research, diagnostic profiling, multiplexed signaling analysis, and high‑content inhibitor discovery.

## Declaration of Generative AI and AI‐Assisted Technologies in the Writing Process

During the preparation of this work, the authors used Gemini (enterprise subscription) to proofread for spelling, grammar, and punctuation. After using this tool, the authors reviewed and edited the content as needed and assume full responsibility for the content of the publication.

## Conflicts of Interest

The authors declare no conflicts of interest.

## Supporting information




**Supporting File**: smll75317‐sup‐0001‐SuppMat.pdf.

## Data Availability

The data that support the findings of this study are openly available in Zenodo at https://doi.org/10.5281/zenodo.18911384, reference number 18911384.
