# Supplementary material for: A Triangulenum‐Based Biosensor Platform for Mapping Kinase Activity in T Cells
Source: Small. 2026 Aug 20;22(58):e75317. doi: 10.1002/smll.75317 (PMC13676840; doi:10.1002/smll.75317)
Supplement: Supplementary file 1 [file SMLL-22-e75317-s001.pdf]

A Triangulenum-Based Biosensor Platform for Mapping Kinase Activity in T Cells

Carmen Salto Girón,[a] Angélica Martín Priego,[a] Francisco Fueyo-González,[b],[c] María Mar Quesada-Moreno,[d] Federico Movilla,[a] Antonio Reinoso,[e] Laura Espinar-Barranco,[c] Rafael Salto,[e] Amparo Navarro,[d] Miguel Fribourg,[c] Angel Orte*[a] and Juan A. González-Vera*[a]

[a] C. Salto Girón, A. Martín Priego, Dr. F. Movilla, Prof. A. Orte,* Dr. J. A. González-Vera*
Nanoscopy-UGR Laboratory. Departamento de Fisicoquímica, Unidad de Excelencia de Química Aplicada a Biomedicina y Medioambiente, Facultad de Farmacia
Universidad de Granada
Campus Cartuja, 18071, Granada, Spain
E-mail: A.O., angelort@ugr.es; JAGV, gonzalezvera@ugr.es

[b] Dr. F. Fueyo-González
Department of Pharmacological Sciences, Translational Transplant Research Center, Immunology Institute
Icahn School of Medicine at Mount Sinai
New York, NY 10029, USA

[c] Dr. F. Fueyo-González, Dr. L. Espinar-Barranco, Dr. M. Fribourg
Department of Medicine, Translational Transplant Research Center, Immunology Institute
Icahn School of Medicine at Mount Sinai
New York, NY 10029, USA

[d] Dr. A. Navarro; Dr. M. M. Quesada-Moreno
Departamento de Química Física y Analítica, Facultad de Ciencias Experimentales
Universidad de Jaén
23071 Jaén, Spain

[e] Prof. R. Salto, A. Reinoso
Departamento de Bioquímica y Biología Molecular II, Unidad de Excelencia de Química Aplicada a Biomedicina y Medioambiente, Facultad de Farmacia
Universidad de Granada
Campus Cartuja, 18071, Granada, Spain

Contents

1. EXPERIMENTAL SECTION **5**

1.1. GENERAL METHODS AND MATERIALS 5
1.2. PREPARATION OF THE TRIANGULENE-BASED BIOSENSORS AND PEPTIDES 5
Table S1. *Peptide sequences and HPLC-MS results of triangulene biosensors.* *6*
1.3. COMPUTATIONAL METHODS 6
1.4. *IN VITRO* LUMINESCENCE EXPERIMENTS 6
1.5. RECOMBINANT ENZYME ASSAY PROTOCOLS 7
1.6. *IN EXTRACTO* ASSAY PROTOCOLS 7
1.7. FLIM MICROSCOPY 8
1.8. ANIMALS, SPLEEN COLLECTION, AND T CELL ISOLATION 9
1.9. T CELL ISOLATION BY NEGATIVE SELECTION 9
1.10. UPTAKE KINETICS AND CELLULAR LOCALIZATION IN T CELLS 10
1.11. FLOW CYTOMETRY EXPERIMENTS 10
1.12. STATISTICAL ANALYSIS 11

2. SUPPLEMENTARY RESULTS **11**

2.1. OPTIMIZATION OF CONDITIONS FOR *IN VITRO* EXPERIMENTS 11
Figure S1. *Fluorescent emission at 560 nm of the Src biosensor hydroxylated (**Src-OH**, black) and phosphorylated (**Src-P**, orange) biosensor at different ATP concentrations (0-1 mM). (A) $MgCl_2$ 1 mM; (B) $MgCl_2$ 5 mM.* *12*
2.2. ADDITIONAL COMPUTATIONAL RESULTS 13
Figure S2. *Molecular orbital energy diagram and representation (isocontour plots 0.02 a.u.) of representative orbitals of **Src-OH**, calculated at the ωB97xD/6-31G* level of theory, indicating the absorption transition from the triangulenium moiety.* *13*
Figure S3. *Molecular orbital energy diagram and representation (isocontour plots 0.02 a.u.) of representative orbitals of phosphorylated **Src-P**, calculated at the ωB97xD/6-31G* level of theory, indicating the absorption transition from the triangulenium moiety.* *14*
Figure S4. *Molecular orbital energy diagram and representation (isocontour plots 0.02 a.u.) of representative orbitals of **Akt1-OH**, calculated at the ωB97xD/6-31G* level of theory, indicating the absorption transition from the triangulenium moiety.* *15*
Figure S5. *Molecular orbital energy diagram and representation (isocontour plots 0.02 a.u.) of representative orbitals of phosphorylated **Akt1-P**, calculated at the ωB97xD/6-31G* level of theory, indicating the absorption transition from the triangulenium moiety.* *16*
Table S2. *Most probable vertical electronic transitions ($\lambda_{vert\text{-}ab}^{calc}$) (in nm and eV in parenthesis), oscillator strength (f), and main components of the $S_0 \rightarrow S_n$ transitions (% contribution) calculated at the TD-ωB97XD/6-31G* level of theory.* *16*
Figure S6. *CREST-extracted structure and subsequently optimized structure, calculated at the ωB97xD/6-31G* level of theory, of **Akt1-OH** and **Akt1-P**, to assess the distance distribution, weighted by the Boltzmann function, between the KU530 and the aromatic side group of Tyr in **Akt1-OH** (orange) and **Akt1-P** (dark blue).* *17*
Figure S7. *CREST-extracted structure and subsequently optimized structure, calculated at the ωB97xD/6-31G* level of theory, of **Akt1-OH** and **Akt1-P**, to assess the distance distribution, weighted by the Boltzmann function, between the two His residues in **Akt1-OH** (magenta) and **Akt1-P** (light pink).* *17*
2.3. SUPPLEMENTARY *IN VITRO* EXPERIMENTS 18

Figure S8. *A and C) Fluorescence emission intensity at 560 nm of unphosphorylated (**Src-OH** and **Akt1-OH**) and phosphorylated peptides (**Src-P** and **Akt1-P**) spectra at different pH values, obtained using mixtures of NaH_2PO_4, Na_2HPO_4, and Na_3PO_4 (10 mM total phosphate concentration). The peptide concentration was 5 μM. λ_{ex} = 530 nm. Error bars represent s.d. from 3 independent measurements. B and D) Relative intensity values normalized to the corresponding value of each peptide at pH 4.4.* *18*
Figure S9. *Michaelis-Menten kinetics with recombinant enzyme. Kinetic profiles used to determine initial phosphorylation velocity for the (A) **Src-OH** biosensor, with 100 ng recombinant kinase; and (B) **Akt1-OH** biosensor, with 50 ng recombinant kinase.* *18*
Figure S10*. Endpoint in extracto assays. Relative emission intensity of the biosensors (**Src-OH**, A; and **Akt1-OH**, B) in the presence of different amounts of cells extracts (Ext): (A) 0.5–40 μg of HeLa-membrane extract for Src; (B) and 0.5–20 μg of HeLa-cytoplasmic extract for Akt1. Each biosensor concentration was fixed at 5 μM. The fluorescence emission at 560 nm (λ_{ex} = 530 nm) was normalized to the value of the unphosphorylated biosensors in buffer PBS pH 7.4 NaCl 100 mM (gray bars). Error bars represent s.d. from 3 independent experiments.* *19*
Figure S11. *Endpoint extract assays for endogenous kinase activity of Src (A) and Akt1 (B) were performed using the peptide biosensors. The biosensor concentration was fixed at 5 μM in all assays. Emission intensity values were normalized to the phosphorylated biosensors in the presence of standard cell extracts (dark-colored bars). The signals of the **Src-OH** (dark orange) and **Akt1-OH** (dark blue) biosensors decreased with increasing concentrations of their respective inhibitors (light-colored bars), reaching the same levels as the unphosphorylated controls (grey bars), at 50 nM Das or 10 μM LY. (A) 10 μg of HeLa-membrane extract (Ext) with 0–200 nM of Dasatinib (Das). (B) 10 μg of HeLa-cytoplasmic extract (Ext) with 0–20 μM of LY294002 (LY). Fluorescence emission at 560 nm (λ_{ex} = 530 nm). Error bars represent s.d. from 3 independent experiments.* *19*
Figure S12. *Endpoint extract assays for endogenous kinase activity using the Pep1-modified biosensors (**Src-Pep1-OH** 5 μM, A; and **Akt1-Pep1-OH** 5 μM, B). Emission intensity values were normalized to the unphosphorylated biosensors in buffer PBS pH 7.4 NaCl 100 mM (grey bars). The emission intensity of the probes increased in the presence of 10 μg standard cell extracts (dark-colored bars; **Src-Pep1-OH**, dark orange; and **Akt1-Pep1-OH**, dark blue). The signal decreased with increasing concentrations of their respective inhibitors, 50 nM Das or 10 μM LY, respectively (light-colored bars). Fluorescence emission at 560 nm (λ_{ex} = 530 nm). Error bars represent s.d. from 3 independent experiments.* *20*
2.4. VIABILITY ASSAYS 20
Figure S13. *Viability test by flow cytometry. Cytotoxicity profile of the **Akt1-Pep-OH** biosensor in mouse primary T cells after 24 h incubation at increasing concentrations (0–100 μM), measured by flow cytometry. Data are presented as mean ± s.d. from three independent experiments.* *21*
2.5. BIOSENSOR CELL UPTAKE 21
Figure S14. *Time-dependent cellular uptake kinetics. Cells were incubated with the **Akt1-Pep1-OH** biosensor (25 μM), and uptake was measured as normalized biosensor emission relative to untreated cells over time. Data are presented as mean ± SD (n = 3). The solid red line represents a nonlinear fit to a one-phase exponential association model, indicating a saturable uptake process.* *22*
2.6. ADDITIONAL IMAGING FIGURES 23
Figure S15. *Additional TG-intensity (A) and τ_{FL} imaging (B) of **Src-Pep1-OH/Ala** peptides in HeLa cells. i) Untreated HeLa cells; ii) cells incubated with Src-Pep1-KU530-Ala; iii) cells incubated with **Src-Pep1-OH**; iv) cells pretreated with 40 nM Das and incubated with **Src-Pep1-OH**. The peptide concentration was 5 μM in all cases. Scale bars correspond to 5 μm scale.* *23*
Figure S16. *Additional TG-intensity (A) and τ_{FL} imaging (B) of **Akt1-Pep1-OH/Ala** peptides in HeLa cells. i) Untreated HeLa cells; ii) cells incubated with **Akt1-Pep1-Ala**; iii) cells incubated with **Akt1-Pep1-OH**; iv) cells pretreated with 10 μM LY294002 and incubated with **Akt1-Pep1-OH**. The peptide concentration was 5 μM in all cases. Scale bars correspond to 5 μm scale.* *24*

3. REFERENCES **25**

4. ANNEX – ADDITIONAL COMPUTATIONAL TABLES **27**

Table S3. *Relative energies of the most stable conformations (< 6 kcal/mol) for the* ***Akt1-OH*** *peptide obtained with the GFN-FF force field implemented in CREST in aqueous medium.* *27*

Table S4. *Relative energies of the most stable conformations (< 6 kcal/mol) for the* ***Akt1-P*** *peptide obtained with the GFN-FF force field implemented in CREST in aqueous medium.* *28*

Table S5. *Relative energies of the most stable conformations (< 6 kcal/mol) for the* ***Src-OH*** *peptide obtained with the GFN-FF force field implemented in CREST in aqueous medium.* *29*

Table S6. *Relative energies of the most stable conformations (< 6 kcal/mol) for the* ***Src-P*** *peptide obtained with the GFN-FF force field implemented in CREST in aqueous medium.* *30*

1. Experimental Section

1.1. General methods and materials

All reagents were of commercial grade with the highest purity and minimal metal content. All the custom peptides were purchased from GL Proteogenix (Schiltigheim, France) and dissolved in doubly deionized water prior use. Reactive maleimide of KU530 triangulenium (KU Dyes, Denmark) was dissolved in DMSO at 2 mM before usage. BioPerformance Certified Phosphate-Buffered Saline (PBS) (10×, SigmaUltra) was diluted to 1× and adjusted to pH 7.5 with NaOH (99.998+%, Aldrich). 5 M NaCl stock solution was prepared by dissolving SigmaUltra sodium chloride in ultrapure water. KREBS buffer at pH 7.4 was prepared with HEPES 30 mM, $NaHCO_3$ 10 mM, NaCl 120 mM, KCl 4 mM, $MgSO_4$ 1 mM, and $CaCl_2$ 1 mM. BSA (Heat Shock Fraction V, Roche) solution was prepared at 20 mg/mL in ultrapure water, filtered through a 0.45 µm syringe filter, and stored at 4 °C. A 0.1% Brij-35 P (Fluka) solution was prepared in doubly deionized water and stored at room temperature. A 7.16 M $MgCl_2$ solution was prepared from magnesium chloride hexahydrate (99.99% trace metals basis, SigmaUltra) in ultrapure water. Mg^{2+} concentration was verified by EDTA titration using Eriochrome Black T as described previously.[1] ATP (adenosine 5′-triphosphate disodium trihydrate, Low Metals Grade, EMD Millipore) was prepared at 10 mM in ultrapure water. Stock solutions of LY294002 (LY) and Dasatinib (Das) were prepared in DMSO and freshly diluted in 50 mM HEPES (pH 7.5) to the desired concentration. Phorbol 12-myristate 13-acetat (PMA) powder was dissolved in 100% DMSO to a high-concentration stock (1 mM), protect from light, aliquot, and store at –20 °C. For cytometry experiments the following reagents were employed: anti-CD4 (PE-Cy7, clone GK1.5, Invitrogen, Cat. No. 25-0041-82), anti-CD8α (Pacific Blue™, clone 53-6.7, Invitrogen, Cat. No. 48-0081-82), fixable viability dye (APC-Cy7, Invitrogen, eFluor™ 780 Fixable Viability Dye, Cat. No. 65-0865-14).

1.2. Preparation of the triangulene-based biosensors and peptides

We labeled peptide-based biosensors incorporating kinase-specific substrate sequences for their respective target kinases (Akt1 or Src), along with their synthetic phosphorylated counterparts, and the non-phosphorylatable controls mutated with an Ala residue, with triangulenium KU530 via maleimide-cysteine bioconjugation. The variants carrying the cell-penetrating peptide Pep1[2] at the *N*-terminus were also labeled. Table S1 provides a comprehensive list of all the sequences used in this study.

Labeling was achieved by reacting peptides (1 mM), each containing a single cysteine residue positioned either +2 or −2 relative to the phosphorylatable site (Ser or Tyr), with KU530-maleimide (1 equivalent). The samples were shacked at room temperature under dark conditions overnight. Labeled peptides were purified from unreacted dye using NAP-5 columns (GE Healthcare) with 1×PBS buffer (pH 7.5) as the eluent.

The concentration of purified peptide-based biosensors was determined using a NanoDrop 2000 spectrophotometer (Thermo Scientific). Each sample was measured three times at 280 nm (Trp absorption) and at 530 nm (KU530 absorption), and the average value was used to calculate concentration based on the molar absorption coefficients: KU530 (ε_{530} = 9,800 $M^{-1}\cdot cm^{-1}$ at 530 nm). Purified peptide stocks were stored at 4 °C for up to 6 months or at −20 °C for extended periods.

Peptide biosensors were analyzed by high-performance liquid chromatography coupled to tandem mass spectrometry (HPLC-MS) using a Waters XEVO TQ-XS system equipped with an electrospray ionization (ESI) source operating in positive mode. Chromatographic separation was performed on a

Sunfire C18 column (4.6 × 50 mm, 3.5 μm) maintained at 30 °C. The mobile phase consisted of solvent A (0.1% formic acid in water) and solvent B (0.1% formic acid in acetonitrile), applied as a gradient at a flow rate of 1 mL/min. Mass spectrometric detection was carried out under optimized conditions for peptide analysis, monitoring the expected m/z values for labeled and unlabeled species. Data acquisition and processing were performed using Waters MassLynx software. The identity and purity of the biosensors were confirmed by retention time, MS/MS fragmentation patterns, and comparison with theoretical masses (Table S1).

Table S1. Peptide sequences and HPLC-MS results of triangulene biosensors.

Peptide	Peptide Sequence[a]	Mol. Formula	HPLC t_R /min[b]	[M] Calcd.	$[M+H]^+$ found[c]	$[M+H]^+$ peak[c]
**Akt1-OH**	Ac-ARKRERAYS*FC(KU530)HHA-CONH2	$C_{103}H_{136}N_{31}O_{23}S^+$	1.91	2208.11	442.6	$[M+5H]^{5+}$
**Akt1-P**	Ac-ARKRERAYpS*FC(KU530)HHA-CONH2	$C_{103}H_{137}N_{31}O_{26}PS^+$	1.79	2286.08	458.2	$[M+5H]^{5+}$
**Akt1-Pep1-OH**	Ac-KETWWETWWTEKK-ARKRERAYS*FC(KU530)HHA-CONH2	$C_{193}H_{256}N_{51}O_{44}S^+$	3.24	4026.54	576.2	$[M+7H]^{7+}$
**Akt1-Pep1-Ala**	Ac-KETWWETWWTEKK-ARKRERAYA*FC(KU530)HHA-CONH2	$C_{192}H_{254}N_{51}O_{44}S+$	2.23	4012.15	669.7	$[M+6H]^{6+}$
**Src-OH**	Ac-EEC(KU530)IY*GEIEA-CONH2	$C_{78}H_{96}N_{13}O_{24}S^+$	3.89	1631.4	331.7	$[M+Na+5H]^{6+}$
**Src-P**	Ac-EEC(KU530)IpY*GEIEA-CONH2	$C_{78}H_{97}N_{13}O_{27}PS^+$	4.35	1711.4	347.7	$[M+Na+5H]^{6+}$
**Src-Pep1-OH**	Ac-KETWWETWWTEKK-EEC(KU530)IY*GEIEA-CONH2	$C_{167}H_{214}N_{33}O_{46}S^+$	2.78	3451.79	863.9	$[M+4H]^{4+}$
**Src-Pep1-Ala**	Ac-KETWWETWWTEKK-EEC(KU530)IA*GEIEA-CONH2	$C_{161}H_{210}N_{33}O_{45}S^+$	2.76	3359.7	840.9	$[M+4H]^{4+}$

[a] In the peptide sequences, the reporter fluorophore KU530 is placed in brackets after the corresponding Cys (C) residue used for labelling; the asterisk (*) indicates the phosphorylatable Tyr (Y) or Ser (S), and the lowercase ‘p’ indicates the phosphorylated state. The N-terminus is acetylated for maintaining similarity with the actual substrate. [b] Reported retention times (t_R) and HPLC conditions are from analytical runs. A flow rate of 5 mL/min and a gradient of 0% →100 % of B over 3 min was used. [c] The data was collected on a mass spectrometer with a triple quadrupole analyzer, WATERS model XEVO TQ-XS.

1.3. Computational methods

An initial conformational search of the peptides **Akt1-OH**, **Akt1-P**, **Src-OH**, and **Src-P** was performed in water using the CREST software[3] and GFN-FF force field.[4] The molecular geometry of the ground state was optimized using the Gaussian 16 program package (version C.01)[5] with the ωB97xD[6] functional in combination with 6-31G* basis set. See CREST tables in the Annex (Tables S3-S6).

1.4. *In vitro* luminescence experiments

Fluorescence intensity spectra of phosphopeptides **Akt1-P** and **Src-P** (5 μM), along with their corresponding unphosphorylated biosensors **Akt1-OH** and **Src-OH** (5 μM), were recorded at 25 °C in 1× PBS buffer (pH 7.5) supplemented with 100 mM NaCl. Fluorescence emission spectra were performed in parallel using a cuvette and a Jasco FP-8300 spectrofluorometer (Jasco, Tokyo, Japan), or in a plate and a CLARIOstar® spectrofluorimeter (BMG LABTECH), to demonstrate the robustness

and reproducibility of the biosensors. Spectra on the Jasco FP-8300 spectrofluorometer were recorded using an excitation wavelength of 530 nm, with both excitation and emission bandwidths set to 5 nm, 1 nm intervals, with a scan speed of 500 nm/min, response time of 1 s, and medium sensitivity. Fluorescence measurements on CLARIOstar® spectrofluorimeter were performed on a Greiner 96-well flat-bottom microplate using a top optic configuration at 25 °C. Each well was read with 20 flashes after double-orbital shaking at 300 rpm for 10 s with a settling time of 0.5 s. Excitation was set at 530 nm (bandwidth 8 nm) and emission at 560 nm (bandwidth 8 nm), with a manually set gain of 2000 and a focal height of 8 mm. Data analysis was carried out using OriginLab 2018 software, and the coefficient between the maximum emission of the biosensor -P and biosensor -OH was calculated. Reported values represent the average of three independent experiments from both instruments.

Fluorescence lifetime measurements were carried out using a FluoTime spectrometer (PicoQuant, Berlin, Germany) equipped with a time-correlated single-photon counting (TCSPC) system. Samples were excited using a pulsed diode laser at 530 nm with a repetition rate of 10 MHz. Emission was collected through a monochromator set to 560 ± 10 nm and detected with a photomultiplier tube. The instrument response function (IRF) was recorded using a Ludox suspension under identical conditions for deconvolution. Data were analyzed using PicoQuant's EasyTau software by fitting to a multi-exponential decay model, and the quality of the fit was assessed by the χ^2 value and residuals. Measurements were performed at 25 °C in PBS, pH 7.4. Amplitude-weighted average lifetimes are reported in Table 1 in the main text.

1.5. Recombinant enzyme assay protocols

Each *in vitro* kinase reaction was carried out in a cuvette with a total volume of 120 µL containing 1× PBS (pH 7.4), 5 mM $MgCl_2$, 0.5 mM ATP, 1 mM DTT, 0.2 mg/mL BSA, 1% glycerol, and 0.1% Brij-35, and incubated at 30 °C. The peptide biosensors (**Src-OH** or **Akt1-OH**) were tested at different concentrations: 0.25, 0.5, 0.75, 1, 2.5, 5, 7, 10, and 20 µM. Kinase amounts were used according to the manufacturer's protocol for recombinant enzymes (Sigma-Ultra), corresponding to 50 ng for Akt1 and 100 ng for Src. Both enzymes were diluted in buffer containing 20 mM HEPES (pH 7.4), 0.01% Brij-35, 5% glycerol, 1 mM DTT, and 1 mg/mL BSA, aliquoted, and stored at −80 °C prior use.

Fluorescence spectra were recorded using the Jasco FP-8300 spectrofluorometer performing in kinetics mode to acquire full spectra every 30 s for 30 min. To define the initial velocity phase, a linear regression analysis was performed by progressively reducing the number of data points. The time interval that yielded the highest coefficient of determination (R^2) was selected as the kinase-specific standard reaction time, which corresponded approximately to the first 5 minutes of the assay. For estimation of maximal velocity (V_{max}) and Michaelis constant (K_M), initial velocity values were plotted against biosensor concentration and fitted to the Michaelis–Menten model using GraphPad Prism 8 (GraphPad Software, La Jolla, CA, USA). According to the Michaelis–Menten curve, K_M was calculated as the substrate concentration required to reach half of V_{max}.

1.6. *In extracto* assay protocols

HeLa cells were selected as the model system for both kinases, Akt1 (cytoplasmic) and Src (membrane), based on their enrichment and subcellular localization, as reported by the Human Protein Atlas.[7-8] The cell line was provided by the Cell Culture Facility at the University of Granada (Spain).

Cells were cultured in DMEM GlutaMAX™ medium (Gibco) supplemented with 10% fetal bovine serum (FBS; Sigma), 100 U/mL penicillin, and 100 µg/mL streptomycin (Sigma). Cultures were maintained at 37 °C in a humidified atmosphere containing 5% CO_2.

For the preparation of cytoplasmic extracts, cells were lysed in RIPA buffer (30 mM Tris-HCl, pH 7.4; 25 mM NaCl; 10 mM NaF; 1 mM sodium pyrophosphate; 1 mM sodium orthovanadate; 1 mM PMSF). Lysates were sonicated and centrifuged at 13,000 rpm for 10 min to remove membranous organelles. For cell membrane extracts, cells were homogenized in M buffer (20 mM HEPES, pH 7.4; 5 mM NaN_3; 250 mM sucrose; 200 µM PMSF) using a Potter homogenizer. Fractionation was performed by differential centrifugation: nuclei (5 min at 800 g), mitochondria (10 min at 9,000 g), and membranes (1 h at 31,000 g). All reagents were purchased from SigmaUltra and dissolved in ultrapure water. Protein concentrations were determined spectrophotometrically at 280 nm. Extracts were aliquoted and stored at −80 °C.

Endpoint kinase assays were conducted in 96-well plates within a thermostated chamber (ClarioStar, BMG) at 25 °C. Each reaction was performed in 120 µL of PBS (1×, pH 7.5) containing 100 mM NaCl, supplemented with 5 mM $MgCl_2$ and 0.5 mM ATP. The use of a plate reader spectrophotometer enabled rapid screening and optimization of assay conditions.

The biosensor concentration was fixed at 5 µM, while increasing amounts of cell extract were added: 0.5–40 µg of HeLa-membrane for Src, and 0.5–20 µg of HeLa-cytoplasm for Akt1. After 30 minutes of incubation, luminescence changes were recorded at 560 nm (λ_{ex} = 530 nm) and compared with a non-phosphorylatable control, on a ClarioSTAR plate reader. All experiments were carried out in triplicate, and error bars represent the standard deviation of the mean. For both kinases, 10 µg of cell extract yielded the greatest fluorescence enhancement without reaching saturation, providing sufficient dynamic range for detecting modulators (1.2x-fold relative to control in both kinases) (Figure S8).

The assays with kinase inhibitors (LY294002, 1–20 µM; Dasatinib, 25–200 nM) were performed following the same protocol, with the cell extract concentration fixed at 10 µg for both Src and Akt1. The inhibitors were added to cell cultures at 60–70% confluency and incubated for 24 h prior to perform the recovery of the cell extracts. The choice of 10 µg of cell extract was based on its ability to provide an optimal balance between signal intensity and assay reliability. At this concentration, kinase activity produced a robust and reproducible fluorescence enhancement without reaching saturation or introducing nonspecific effects that can occur at higher protein loads. This condition was therefore selected as the standard for subsequent assays, as it maximized sensitivity and reproducibility under physiologically relevant conditions.

1.7. FLIM microscopy

HeLa cell lines were used in the live-cell imaging experiments. Cells were cultured at 37 °C in Dulbecco's Modified Eagle Medium (DMEM) containing 10% fetal bovine serum, 2 mM glutamine, and standard concentrations of penicillin (100 U/mL) and streptomycin (0.1 mg/mL). For microscopy, cells were plated in 8-well iBIDI chambers at 1.6 × 10^5 cells per well and allowed to grow for 24 h to reach approximately 80% confluence.

Before imaging, kinase biosensor or control peptides (5 µM; **Src-Pep1-OH**, **Akt1-Pep1-OH**, **Src-Pep1-Ala**, or **Akt1-Pep1-Ala** variants) were mixed with the cell-penetrating peptide Pep1 (10 µM) in PBS (pH 7.4, 0.9% NaCl) for 1 h to allow forming assemblies designed to enhance cellular uptake.[2, 9] Cell culture medium was removed, and the wells were washed twice with PBS. When inhibitor treatments were performed, cells were incubated for at least 2 h with the appropriate compound (LY 294002, 10 µM, for Akt1; Dasatinib, 40 nM, for Src). Biosensor–Pep1 complexes (5 µM) were then

added to the cells and incubated for 30 min under the same buffer conditions, and a final wash with PBS was done.

Confocal and FLIM measurements were obtained on an Abberior Expert Line microscope (Abberior Instruments GmbH, Germany). The triangulenium dye KU530 was excited using a 485 nm pulsed laser operated at 5 MHz, providing a 200 ns TCSPC window. Emission was collected through a 605/50 nm band-pass filter using an avalanche photodiode. Standard confocal images covered areas between 40 × 40 μm² and 80 × 80 μm², acquired at 70 nm per pixel with a pixel dwell time of 500 μs.

Fluorescence-lifetime data were processed using SymphoTime 64 (PicoQuant). Fluorescence decays were fitted on a per-pixel basis using a monoexponential fitting with iterative reconvolution and an instrument response function extracted from global decay traces. When sufficient photon counts were available, a biexponential model was applied instead, and an intensity-weighted lifetime image was subsequently calculated. To improve photon statistics, spatial (2×2) and temporal (8-channel) binning were applied. A 5 ns temporal gate was used on the intensity image to suppress autofluorescence and exclude short-lifetime components. As a result, a two-stack image was exported: the time-gated (TG) intensity, I^{TG}, and the average intensity weighted lifetime, τ_{FL}.

Further processing was carried out in Fiji (ImageJ).[10] TG-intensity images were smoothed with a Gaussian filter (σ = 0.6) and colored following a red-hot scale. FLIM images were colored using a rainbow color scale. For quantification, regions of interest (ROIs) were identified in the intensity channel using the *Li* automatic threshold method and applied to the TG-intensity and lifetime images for readouts.

1.8. Animals, spleen collection, and T cell isolation

Male and female C57BL/6 (H-2K^b) mice were purchased from The Jackson Laboratory and bred in the animal facilities of the Icahn School of Medicine at Mount Sinai. Mice were 8 weeks of age at the start of the experiments. Data shown in Figures 8–9 (main text) include both male and female mice, as animals of both sexes were used for the analysis of different T-cell populations. All experiments were performed in strict accordance with the recommendations in the *Guide for the Care and Use of Laboratory Animals* and under a protocol (IACUC-2018-2) approved by the Institutional Animal Care and Use Committee of the Icahn School of Medicine at Mount Sinai. All animals were housed in the animal facilities of the Icahn School of Medicine at Mount Sinai. Spleens were harvested in PBS and mechanically dissociated through a 70 μm mesh strainer using the back of a syringe plunger. Cell suspensions were centrifuged and the supernatant was discarded. Red blood cells were lysed using ACK lysis buffer (2 min at room temperature; Roche). Cells were then centrifuged again, resuspended in PBS, and filtered through a 70 μm nylon mesh to obtain a single-cell suspension. The resulting splenocyte suspensions were used directly for different assays or for the isolation of pan T cells by magnetic negative selection (EasySep™ Mouse T Cell Isolation Kit, STEMCELL Technologies) prior to T-cell culture experiments.

1.9. T cell isolation by negative selection

T cells were isolated from the splenocyte suspension using the EasySep™ Mouse T Cell Isolation Kit (STEMCELL Technologies), following the immunomagnetic negative-selection protocol. Briefly, cells were adjusted to 1 × 10^8 cells/mL, mixed with EasySep™ Mouse FcR Blocker (20 μL/mL), and transferred into 5 mL polystyrene tubes. The EasySep™ Mouse T Cell Isolation Cocktail was then added at 50 μL/mL, mixed gently, and incubated at room temperature for 10 minutes. RapidSpheres™

magnetic particles were vortexed to ensure homogeneity and added to the sample as instructed. Tubes were placed into the EasySep™ magnet, allowing magnetically labeled unwanted cells to be retained while untouched T cells were collected by pouring off the supernatant into a new tube, yielding a highly purified population suitable for immediate downstream applications.

1.10. Uptake kinetics and cellular localization in T cells

Uptake kinetics and intracellular localization in T cells were analyzed by live-cell confocal microscopy using a Zeiss LSM 780 inverted laser-scanning confocal microscope equipped with a 34-channel spectral detector and an on-stage incubation system maintaining 37 °C, 5% CO_2, and controlled humidity. Isolated mouse T cells were plated in glass-bottom imaging dishes and maintained in phenol-red-free imaging medium during acquisition. Fluorescence was detected in the FITC channel using 488 nm laser excitation, and emission was collected within the 500–550 nm spectral window (FITC-equivalent detection range). Laser power and detector gain were minimized to reduce photobleaching and phototoxicity while maintaining adequate signal-to-noise ratios. Time-lapse acquisition was performed with frame intervals of 5 min to monitor uptake kinetics. Cells were imaged immediately after addition of the sensor to visualize rapid internalization and intracellular localization within the first minutes and were followed until fluorescence reached a plateau (~30 min). After the initial 30-min loading period, soluble anti-CD3 and anti-CD28 antibodies (1–5 µg/mL) were added directly to the imaging chamber while time-lapse acquisition continued for an additional 20–30 min. Confocal images were analyzed using Fiji/ImageJ. Background fluorescence was subtracted using regions of interest (ROIs) outside cells, and fluorescence intensities were normalized to the pre-stimulation baseline (F/F_0). For uptake measurements, whole-cell ROIs were used, whereas single-cell ROIs were tracked over time for kinetic analysis. Acquisition and processing parameters were optimized to minimize photobleaching and ensure accurate quantification of dynamic fluorescence changes in live cells.

1.11. Flow cytometry experiments

Mouse splenocytes were loaded with the **Akt1-Pep1-OH** sensor (25 µM, 30 min at 37 °C), washed twice with PBS, and stained on ice with fluorophore-conjugated anti-CD4 (PE-Cy7, clone GK1.5, Invitrogen, Cat. No. 25-0041-82) and anti-CD8α (Pacific Blue™, clone 53-6.7, Invitrogen, Cat. No. 48-0081-82) together with a fixable viability dye (APC-Cy7, Invitrogen, eFluor™ 780 Fixable Viability Dye, Cat. No. 65-0865-14) for 20 min in the dark. After two additional washes, cells were resuspended in acquisition buffer and analyzed by flow cytometry. Samples were acquired on a BD FACSSymphony A5 flow cytometer (BD Biosciences). The **Akt1-Pep1-OH** sensor fluorescence was detected on the FITC channel (488 nm excitation, 530/30 emission filter). Compensation controls and fluorescence-minus-one (FMO) controls were used when required.

Gating strategy. Lymphocytes were identified based on forward- and side-scatter (FSC-A vs SSC-A) parameters. Doublets were excluded using FSC-H versus FSC-A, and dead cells were excluded using the viability dye. T-cell subsets were identified as $CD4^+$ and $CD8^+$ populations, and fluorescence intensity of the **Akt1-Pep1-OH** sensor was quantified within each subset.

Comparative stimulation (αCD3/αCD28 vs PMA). After sensor loading (30 min), cells were washed twice and resuspended in fresh pre-warmed medium. Cells were then stimulated either with soluble anti-CD3 and anti-CD28 antibodies (TCR/co-stimulatory activation) or phorbol 12-myristate 13-acetate (PMA; 50–100 nM) to induce PKC-dependent signaling. Samples were analyzed 30 min after stimulation, and fluorescence changes were monitored over a total period of 20–30 min.

Fluorescence intensity was quantified as median fluorescence intensity (MFI) for each subset and normalized to corresponding unstimulated controls from the same mouse. Data were analyzed using FlowJo (BD Biosciences) or FCS Express 7 (De Novo Software). Acquisition and analysis parameters were kept constant across experimental groups.

1.12. Statistical Analysis

Experimental data underwent specific pre-processing and transformation steps depending on the assay type, which has been described in the previous sections. For *in vitro* and *in extracto* luminescence assays, raw fluorescence emission intensities recorded at 560 nm were normalized to their corresponding unphosphorylated controls or standard cell extracts to determine relative emission ratios and fold-changes. For recombinant enzyme assays, the initial phosphorylation velocity phase was established via linear regression analysis by systematically reducing data points to identify the time window yielding the maximum coefficient of determination. Analysis protocols of time-resolved fluorimetry, confocal microscopy of cellular uptake kinetics, and FLIM microscopy are described in the corresponding sections above. For flow cytometry and cell viability assays, debris was excluded using sequential forward- and side-scatter (FSC-A vs. SSC-A) gating, and doublets were removed via FSC-A vs. FSC-H plots. Fluorescence gating boundaries were defined using fluorescence-minus-one (FMO) controls where applicable, and target signals were quantified as median fluorescence intensities (MFI) normalized to individual, intra-animal unstimulated controls.

Quantitative results are presented as the mean ± standard deviation (s.d.). Independent *in vitro* spectrophotometric experiments, *in extracto* kinase assays, cell loading kinetics, and flow-cytometric viability assessments were performed in triplicate as independent experiments ($n = 3$). For primary T-cell flow cytometry experiments, a minimum of 3×10^4 single-cell events were collected per sample, with biological replicates derived from independent animals ($n = 3$).

FLIM images and TG-intensity images were obtained from two different, independent sets of experiments, collecting a minimum of 8 images in each repetition. Box plots show individual events representing the readout of each individual image. Only images showing clearly dead cells or excessive cell debris were discarded in the quantification. The boxes size represents s.d. while whiskers represent 90% of the population for each condition. The average lifetimes of these populations are reported as the mean ± s.d.

Statistical analyses were performed using GraphPad Prism 10 (GraphPad Software, San Diego, CA, USA). Comparisons between two groups were conducted using an unpaired two-tailed Student's t-test, while comparisons involving more than two groups were analyzed using one-way ANOVA followed by Tukey's multiple-comparison post hoc test. Statistical significance is indicated as $p < 0.05$ (*) and $p < 0.01$ (**).

2. Supplementary results

2.1. Optimization of conditions for *in vitro* experiments

Preliminary optimization assays were performed using the **Src-OH** biosensor and its phosphorylated version to determine the optimal concentrations of ATP and Mg^{2+} for the *in vitro* kinase reaction. Reactions were prepared under standard buffer conditions (1× PBS, pH 7.4), 5 μM of **Src-OH/-P**, while varying ATP and $MgCl_2$ concentrations across a defined range (5-10 mM Mg^{2+} and 0-0.1-0.5-1 mM ATP).[11] The coefficient between the maximum emission (560 nm) of the biosensor -P

and biosensor -OH was calculated for each condition, all the increases were very constant (independently from the ATP concentration), for Mg^{2+} 1 mM was 8.55 ± 0.15, and for Mg^{2+} 5 mM was 9.2 ± 0.05; these demonstrates the robustness of the biosensor design. The combination of 5 mM $MgCl_2$ and 0.5 mM ATP was selected as the standard for subsequent experiments, as it yielded the highest efficiency without biosensor fluorescent emission depletion; also, it is common and highly recommended practice in kinase kinetic assay protocols (recommended concentration ratios of ATP 1:3 to 10 Mg^{2+}).[12] We also checked negligible influence of ATP and $MgCl_2$ in the average lifetime increases in **Src-OH/-P** and **Akt1-OH/-P**.

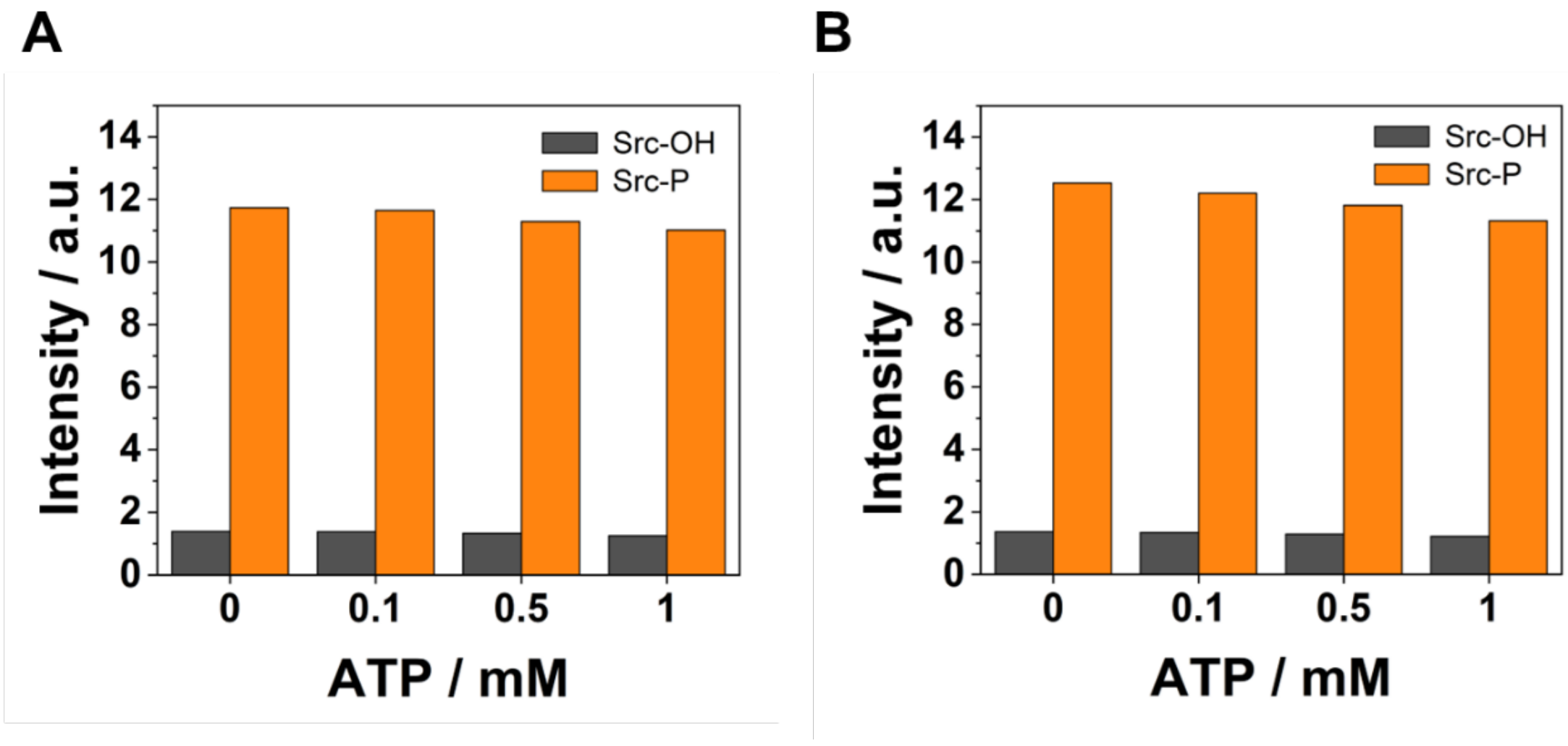

Figure S1. Fluorescent emission at 560 nm of the Src biosensor hydroxylated (**Src-OH**, black) and phosphorylated (**Src-P**, orange) biosensor at different ATP concentrations (0-1 mM). (A) $MgCl_2$ 1 mM; (B) $MgCl_2$ 5 mM.

2.2. Additional Computational Results

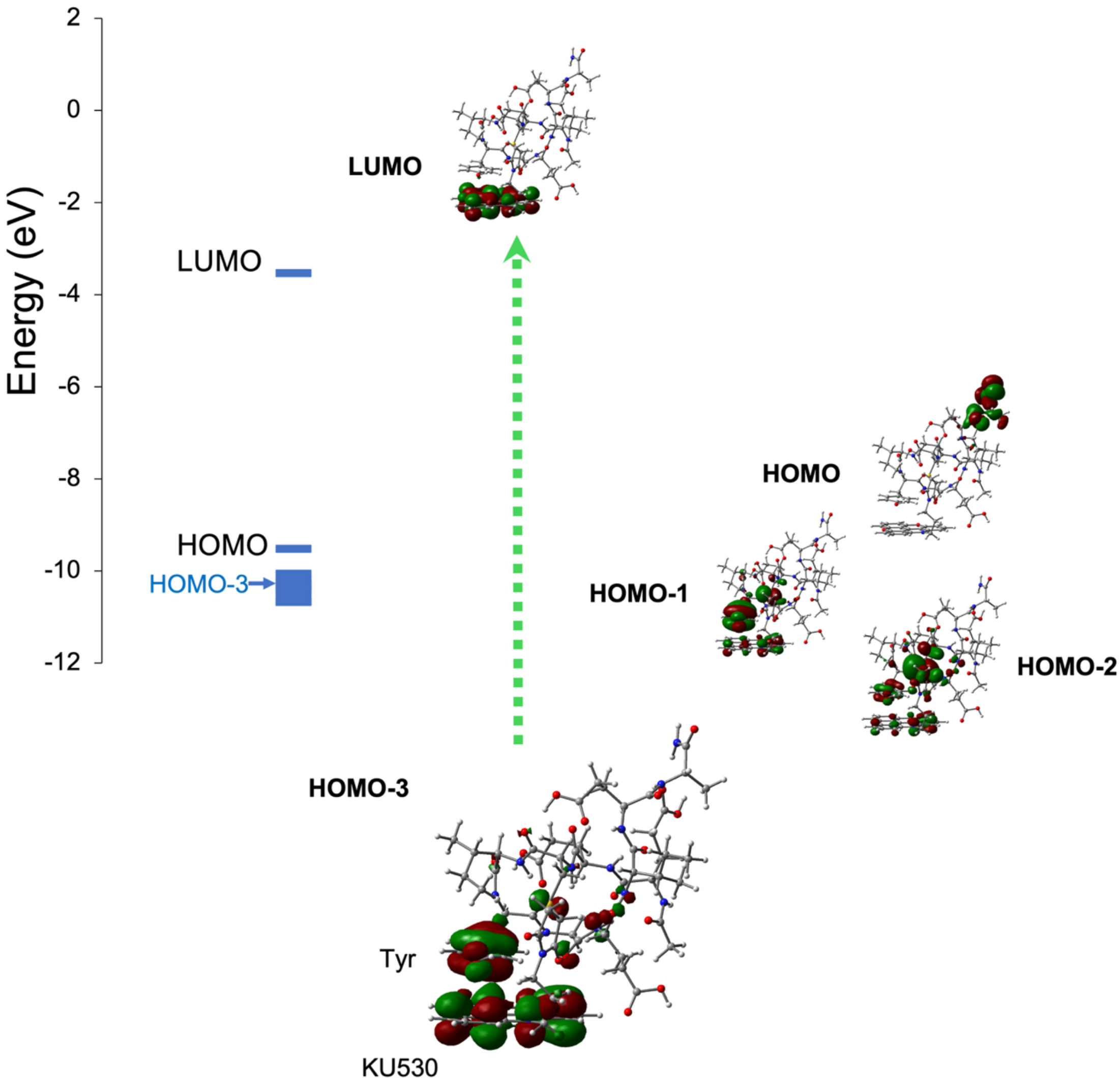

Figure S2. Molecular orbital energy diagram and representation (isocontour plots 0.02 a.u.) of representative orbitals of **Src-OH**, calculated at the ωB97xD/6-31G* level of theory, indicating the absorption transition from the triangulenium moiety.

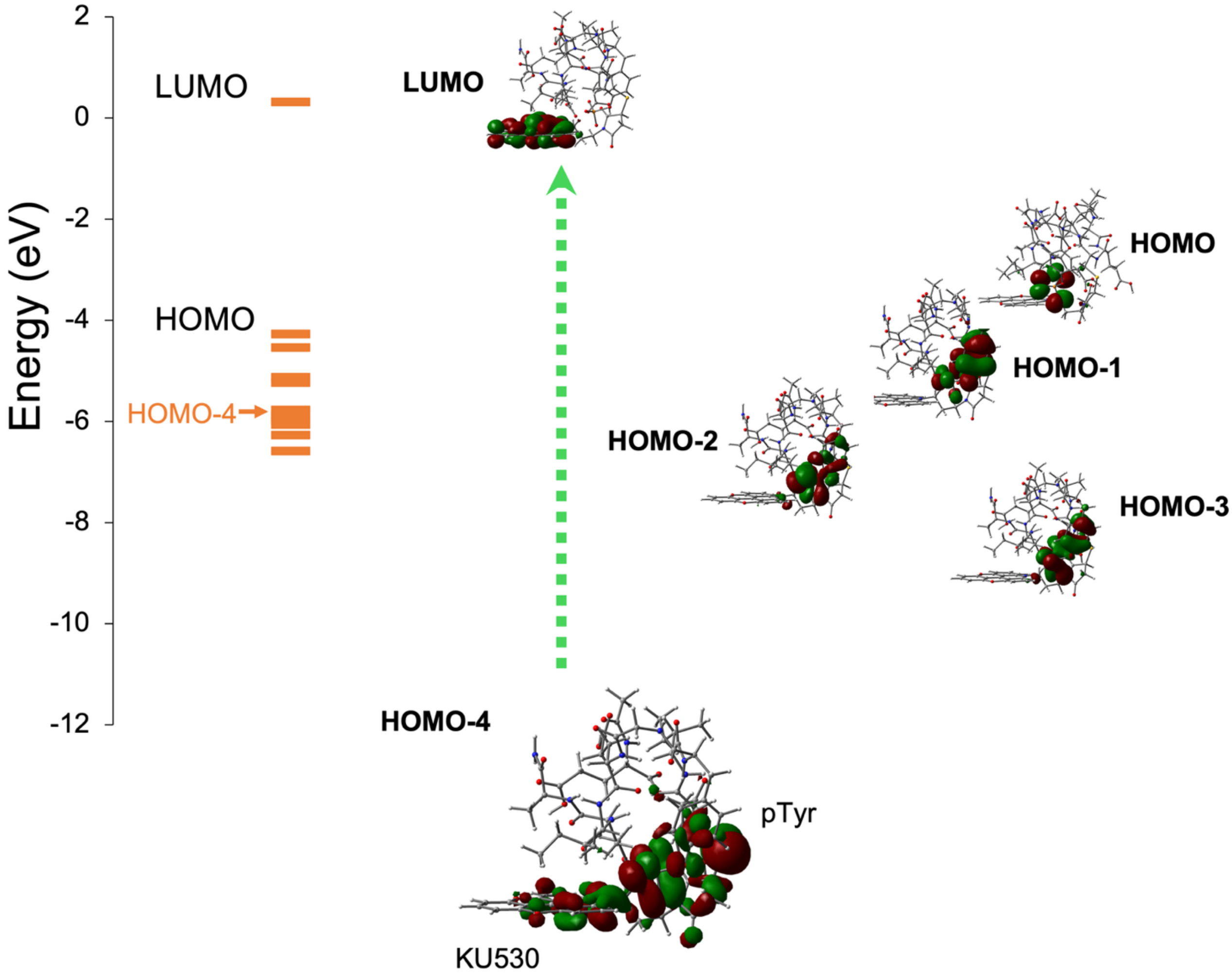

Figure S3. Molecular orbital energy diagram and representation (isocontour plots 0.02 a.u.) of representative orbitals of phosphorylated **Src-P**, calculated at the ωB97xD/6-31G* level of theory, indicating the absorption transition from the triangulenium moiety.

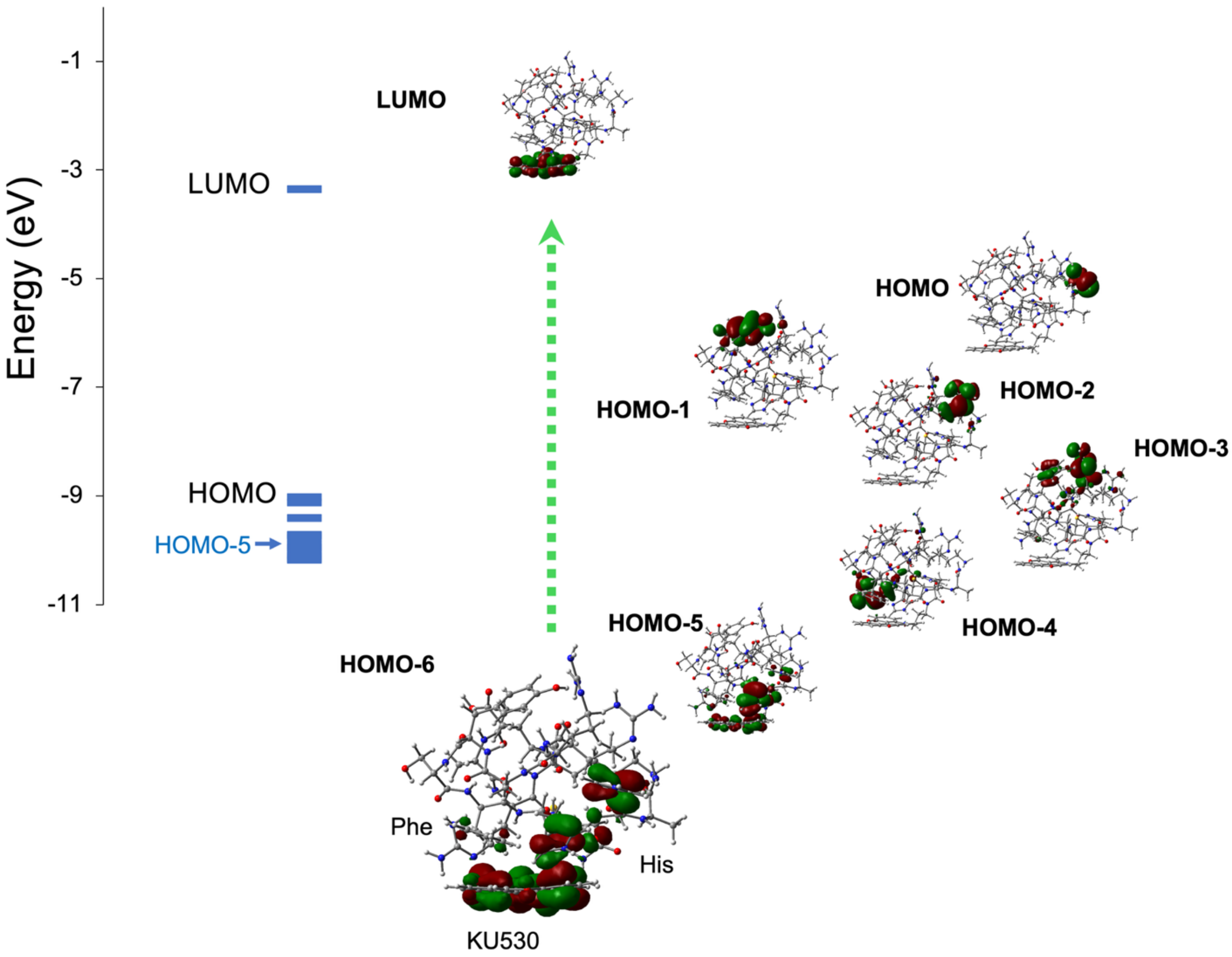

Figure S4. Molecular orbital energy diagram and representation (isocontour plots 0.02 a.u.) of representative orbitals of **Akt1-OH**, calculated at the ωB97xD/6-31G* level of theory, indicating the absorption transition from the triangulenium moiety.

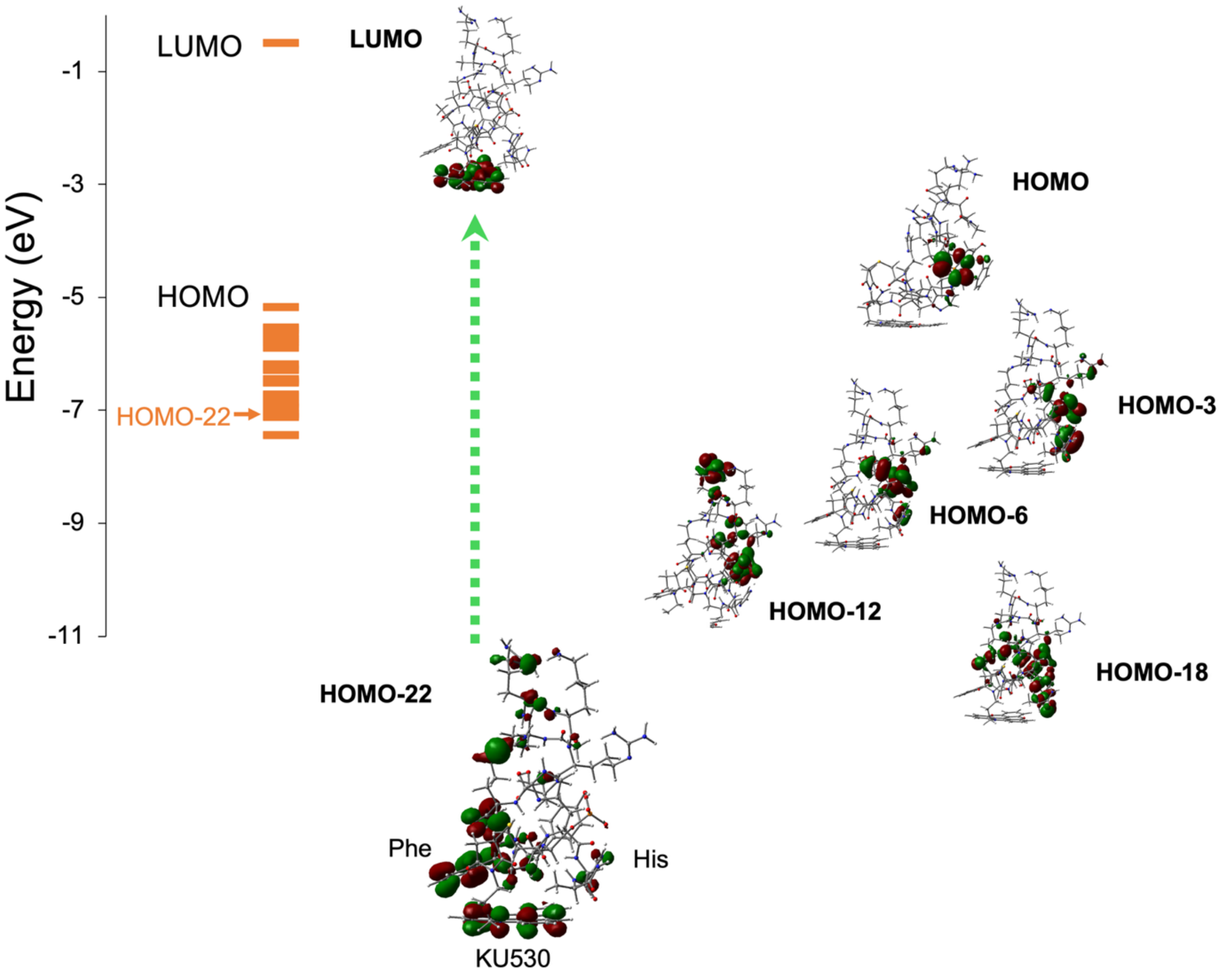

Figure S5. Molecular orbital energy diagram and representation (isocontour plots 0.02 a.u.) of representative orbitals of phosphorylated **Akt1-P**, calculated at the ωB97xD/6-31G* level of theory, indicating the absorption transition from the triangulenium moiety.

Table S2. Most probable vertical electronic transitions ($\lambda_{vert\text{-}ab}^{calc}$) (in nm and eV in parenthesis), oscillator strength (f), and main components of the $S_0 \rightarrow S_n$ transitions (% contribution) calculated at the TD-ωB97XD/6-31G* level of theory.

Peptide	$\lambda_{vert\text{-}ab}^{calc}$ /nm (eV)	f	% Contribution
**Src-OH**	410 (3.02)	0.1200	H–3 → L (51), H–2 → L (16), H–1 → L (30)
**Src-P**	427 (2.90)	0.0965	H–9 → L (14), H–5 → L (13), H–4 → L (20), H–1 → L (31)
**Akt1-OH**	409 (3.04)	0.1348	H–6 → L (64), H–5 → L (25)
**Akt1-P**	399 (3.11)	0.1379	H–26 → L (14), H–25 → L (17), H–24 → L (9), H–23 → L (23), H–22 → L (25)

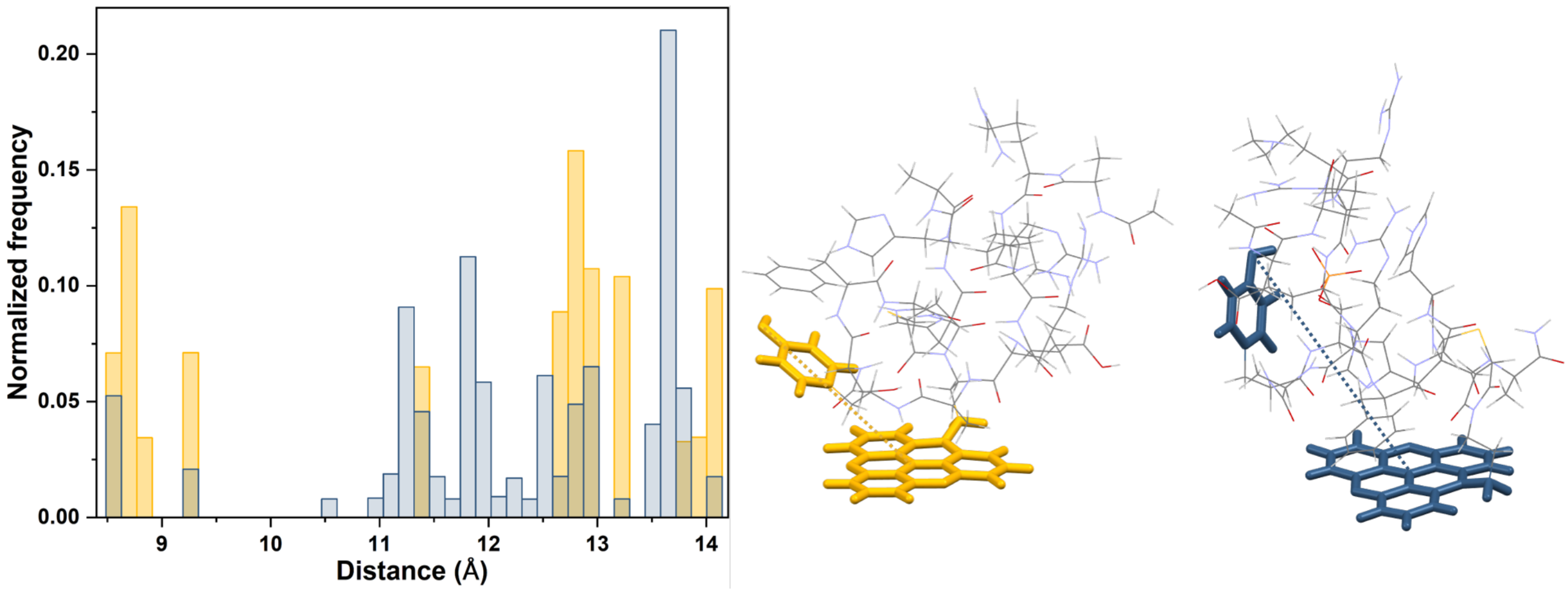

Figure S6. CREST-extracted structure and subsequently optimized structure, calculated at the ωB97xD/6-31G* level of theory, of **Akt1-OH** and **Akt1-P**, to assess the distance distribution, weighted by the Boltzmann function, between the KU530 and the aromatic side group of Tyr in **Akt1-OH** (orange) and **Akt1-P** (dark blue).

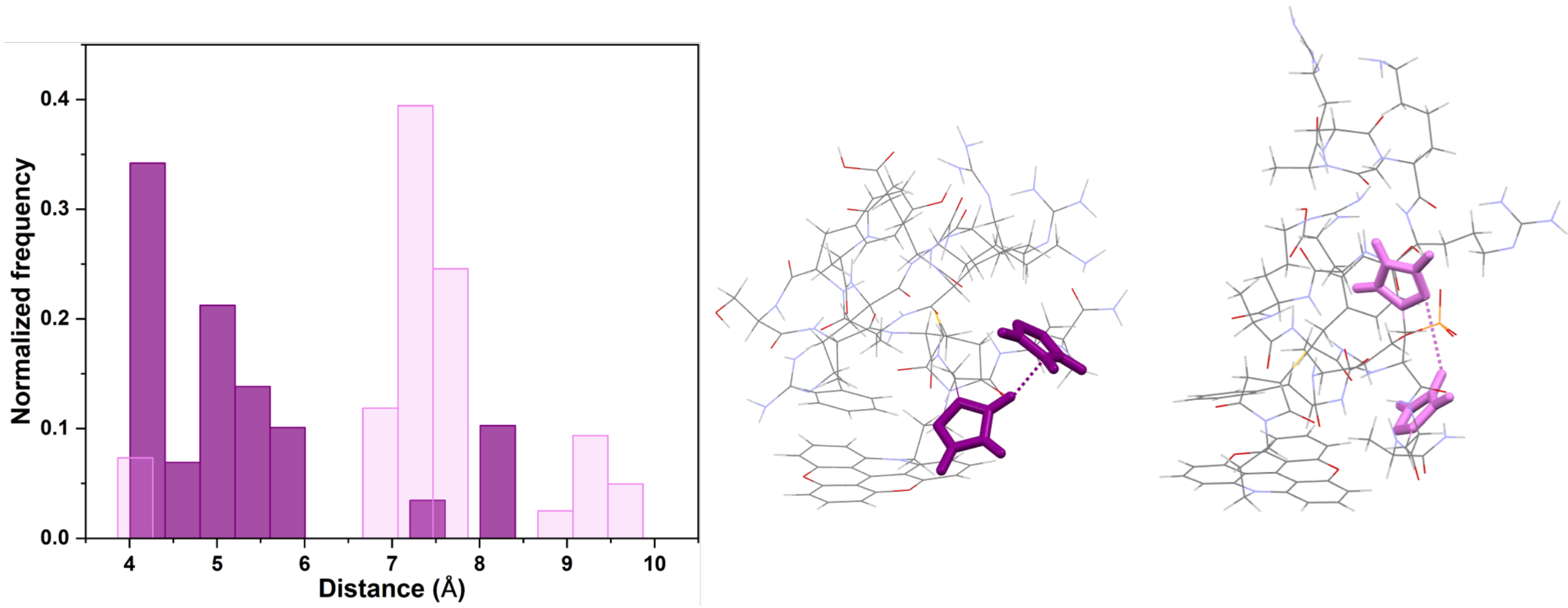

Figure S7. CREST-extracted structure and subsequently optimized structure, calculated at the ωB97xD/6-31G* level of theory, of **Akt1-OH** and **Akt1-P**, to assess the distance distribution, weighted by the Boltzmann function, between the two His residues in **Akt1-OH** (magenta) and **Akt1-P** (light pink).

2.3. Supplementary *in vitro* experiments

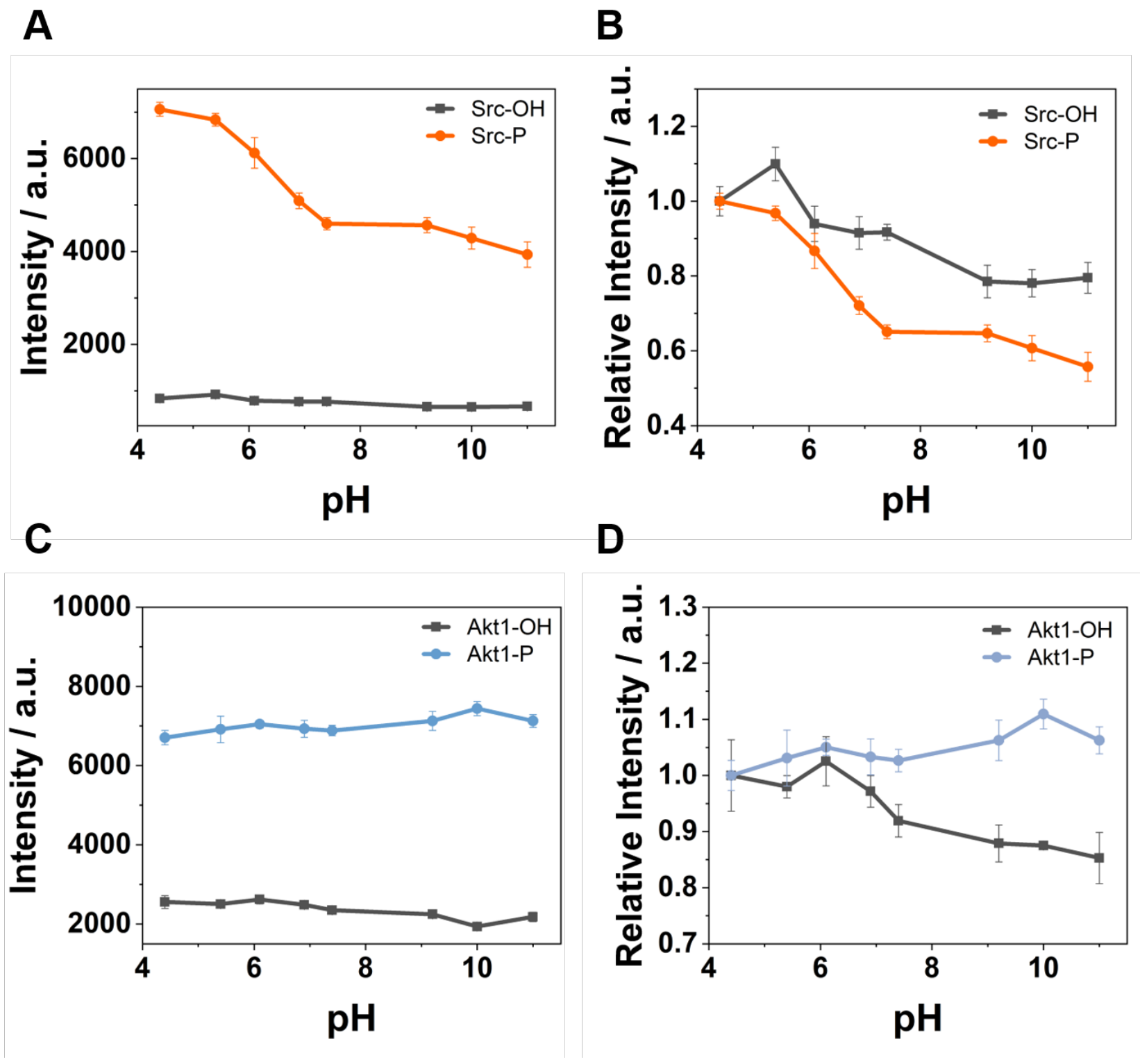

Figure S8. A and C) Fluorescence emission intensity at 560 nm of unphosphorylated (**Src-OH** and **Akt1-OH**) and phosphorylated peptides (**Src-P** and **Akt1-P**) spectra at different pH values, obtained using mixtures of NaH_2PO_4, Na_2HPO_4, and Na_3PO_4 (10 mM total phosphate concentration). The peptide concentration was 5 μM. λ_{ex} = 530 nm. Error bars represent s.d. from 3 independent measurements. B and D) Relative intensity values normalized to the corresponding value of each peptide at pH 4.4.

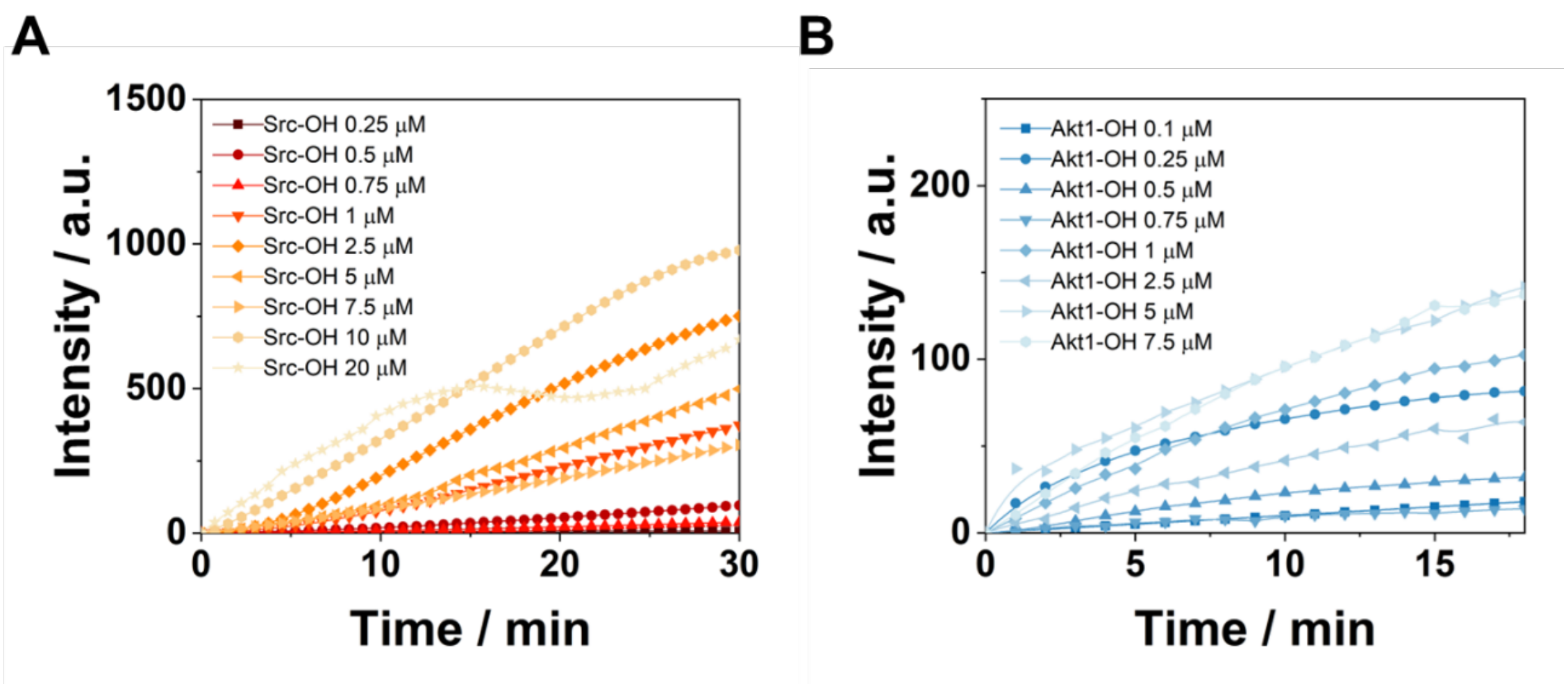

Figure S9. Michaelis-Menten kinetics with recombinant enzyme. Kinetic profiles used to determine initial phosphorylation velocity for the (A) **Src-OH** biosensor, with 100 ng recombinant kinase; and (B) **Akt1-OH** biosensor, with 50 ng recombinant kinase.

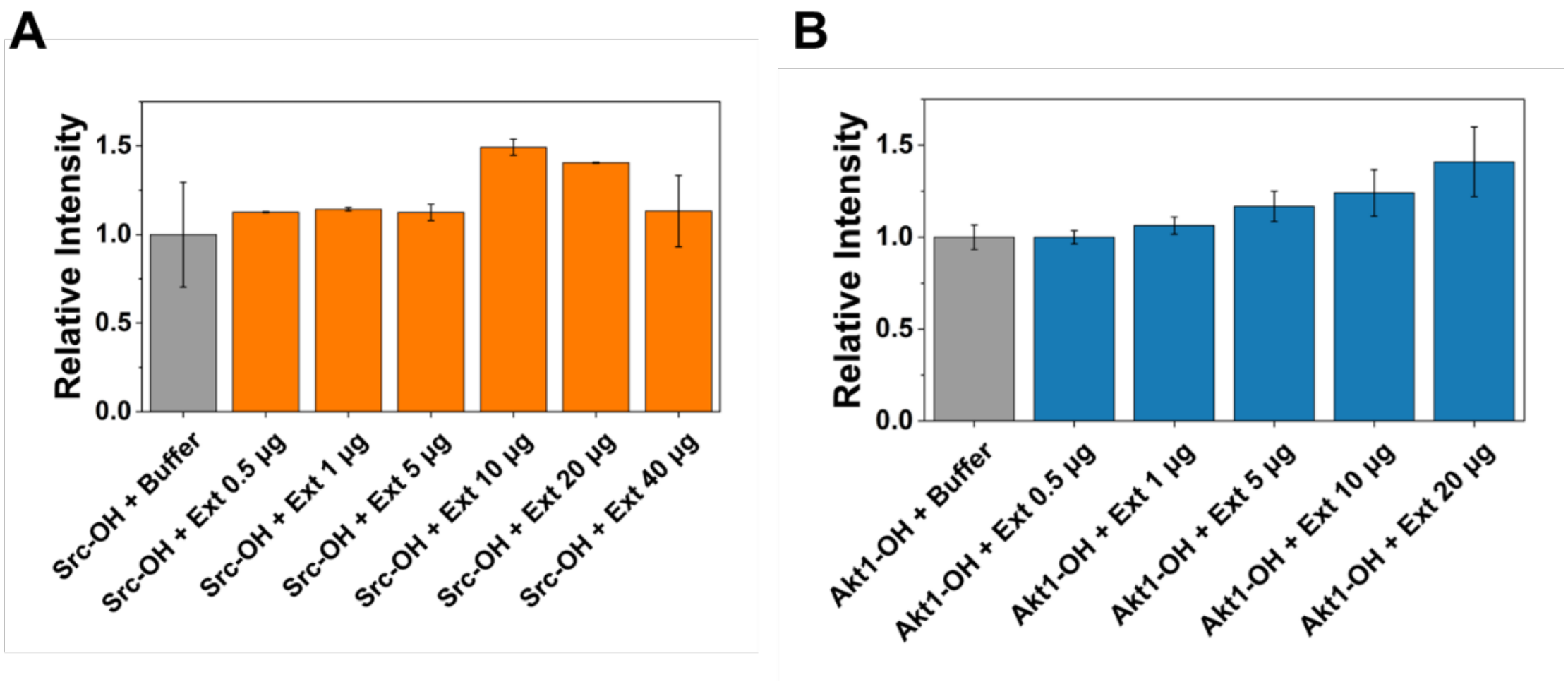

Figure S10. Endpoint *in extracto* assays. Relative emission intensity of the biosensors (**Src-OH**, A; and **Akt1-OH**, B) in the presence of different amounts of cells extracts (Ext): (A) 0.5–40 µg of HeLa-membrane extract for Src; (B) and 0.5–20 µg of HeLa-cytoplasmic extract for Akt1. Each biosensor concentration was fixed at 5 µM. The fluorescence emission at 560 nm (λ_{ex} = 530 nm) was normalized to the value of the unphosphorylated biosensors in buffer PBS pH 7.4 NaCl 100 mM (gray bars). Error bars represent s.d. from 3 independent experiments.

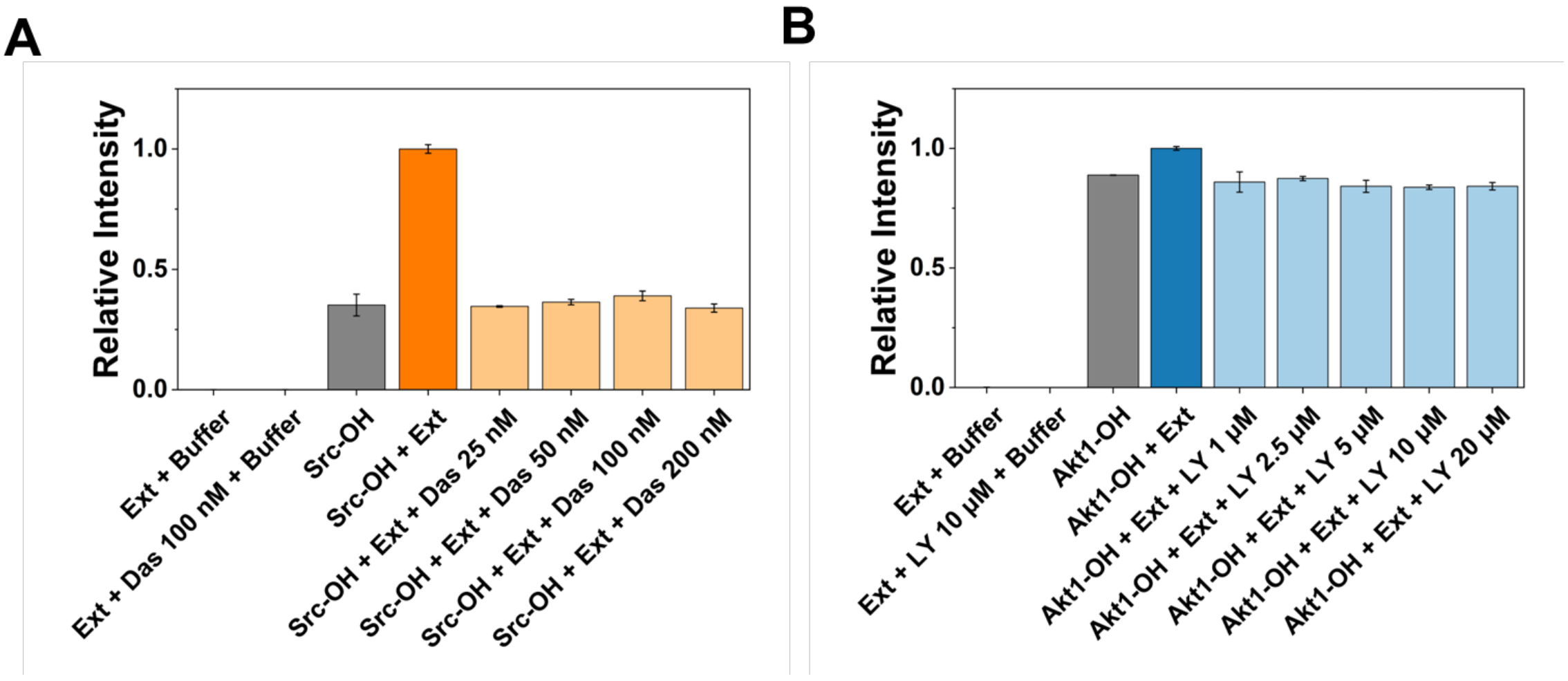

Figure S11. Endpoint extract assays for endogenous kinase activity of Src (A) and Akt1 (B) were performed using the peptide biosensors. The biosensor concentration was fixed at 5 µM in all assays. Emission intensity values were normalized to the phosphorylated biosensors in the presence of standard cell extracts (dark-colored bars). The signals of the **Src-OH** (dark orange) and **Akt1-OH** (dark blue) biosensors decreased with increasing concentrations of their respective inhibitors (light-colored bars), reaching the same levels as the unphosphorylated controls (grey bars), at 50 nM Das or 10 µM LY. (A) 10 µg of HeLa-membrane extract (Ext) with 0–200 nM of Dasatinib (Das). (B) 10 µg of HeLa-cytoplasmic extract (Ext) with 0–20 µM of LY294002 (LY). Fluorescence emission at 560 nm (λ_{ex} = 530 nm). Error bars represent s.d. from 3 independent experiments.

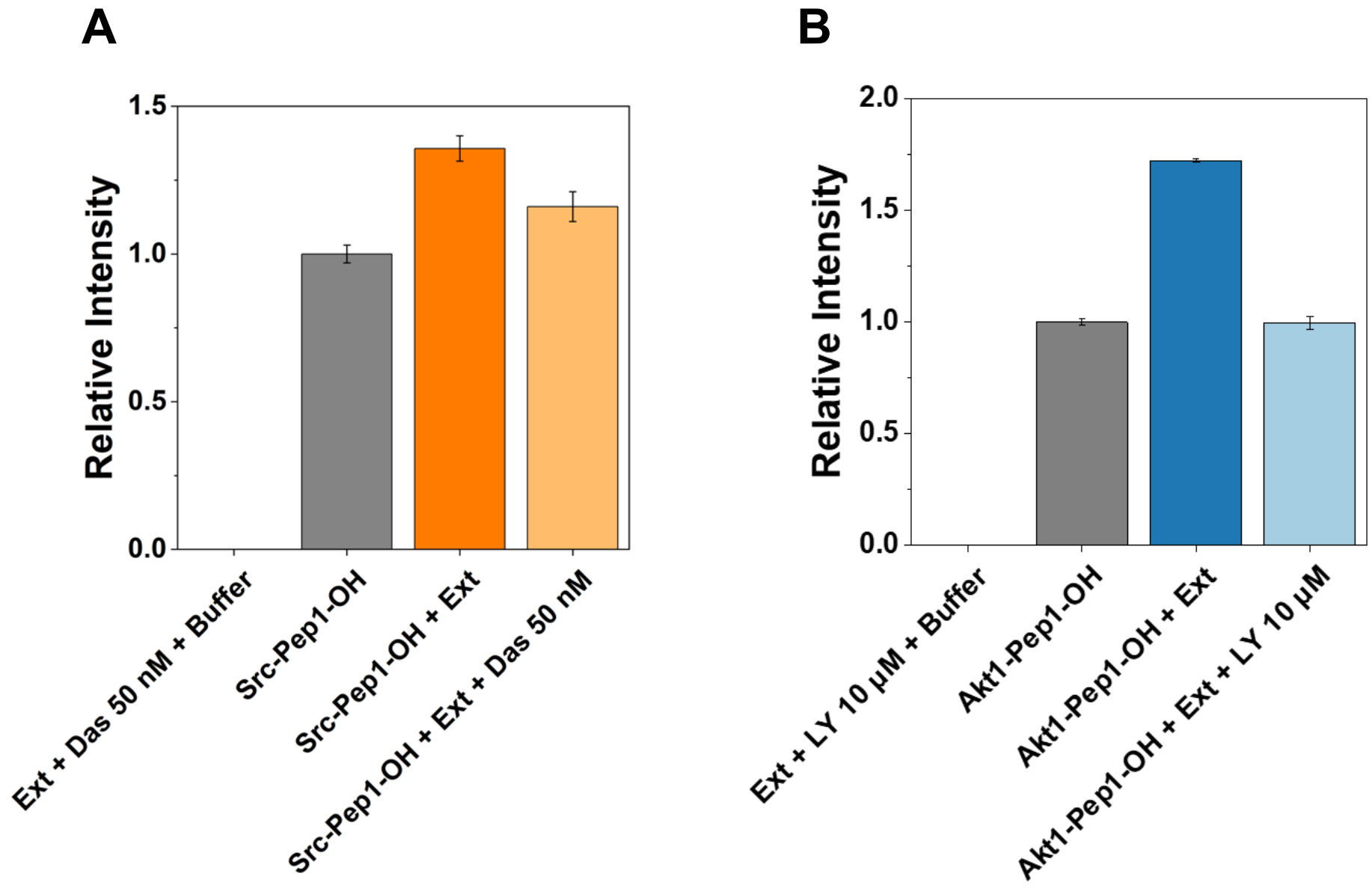

Figure S12. Endpoint extract assays for endogenous kinase activity using the Pep1-modified biosensors (**Src-Pep1-OH** 5 µM, A; and **Akt1-Pep1-OH** 5 µM, B). Emission intensity values were normalized to the unphosphorylated biosensors in buffer PBS pH 7.4 NaCl 100 mM (grey bars). The emission intensity of the probes increased in the presence of 10 µg standard cell extracts (dark-colored bars; **Src-Pep1-OH**, dark orange; and **Akt1-Pep1-OH**, dark blue). The signal decreased with increasing concentrations of their respective inhibitors, 50 nM Das or 10 µM LY, respectively (light-colored bars). Fluorescence emission at 560 nm (λ_{ex} = 530 nm). Error bars represent s.d. from 3 independent experiments.

2.4. Viability assays

Systematic cell viability testing (e.g., MTT) was not performed in each experiment due to three established safeguards: (i) prior validation of similar triangulenium fluorophores in the bibliography, which exhibit negligible cytotoxicity (< 40 µM of triangulenium salt), and preserved functions and morphology;[13-16] (ii) use of Pep-1, a non-covalent delivery vector widely recognized for preserving membrane integrity and protein function, unlike other CPPs that can induce stress responses;[2, 9] and (iii) functional integrity checks, confirmed by the biosensor's ability to report kinase activity and respond to inhibitors, indicating intact signaling pathways and metabolic competence.[17] Together, these factors provide strong support for cell viability at the concentrations employed.

Nonetheless, a preliminary viability assay was conducted in mouse primary T cells for the flow cytometry experiments (see isolation procedure from splenocytes in the Experimental section). **Akt1-Pep1-OH** was pre-incubated with Pep1 at a 1:2 ratio for 1 hour and added to the cells for 24 hours at increasing concentrations of the pre-coordinated biosensor (0–100 µM). Following incubation, Fixable Viability Dye eFluor™ 450 (Fisher Scientific; 1 µL/mL) was added for 20 min at 4 °C. Samples were analyzed on a BD FACS Symphony A5 flow cytometer (BD Biosciences), and data were acquired using BD FACSDiva software. Fluorescence excitation was performed using a 405 nm violet laser for Pacific Blue, along with additional lasers depending on the experimental panel. Pacific Blue–conjugated antibodies were excited with the 405 nm laser, and emission was collected using a 450/40 nm band-pass filter, corresponding to the standard Pacific Blue/BV421 detection channel. Cells were gated sequentially using FSC and SSC to exclude debris, followed by doublet discrimination via FSC-A vs FSC-H (single cell selection). Fluorescence gates were defined using fluorescence-minus-one (FMO) controls when applicable. A minimum of 3×10^4 events were collected per sample. Data

analysis was performed using FlowJo (version 10.10, BD Biosciences). Results are reported as the percentage of viability when comparing the median fluorescence intensity (MFI) of the Fixable Viability Dye eFluor450 (Pacific Blue channel), as specified in each experiment.

As shown in Fig. S11, the sensor displayed no measurable toxicity up to 25 μM. At concentrations above 50 μM, a slight decrease in cell viability was observed, potentially due to partial inhibition of Akt1 kinase activity by the sensor.

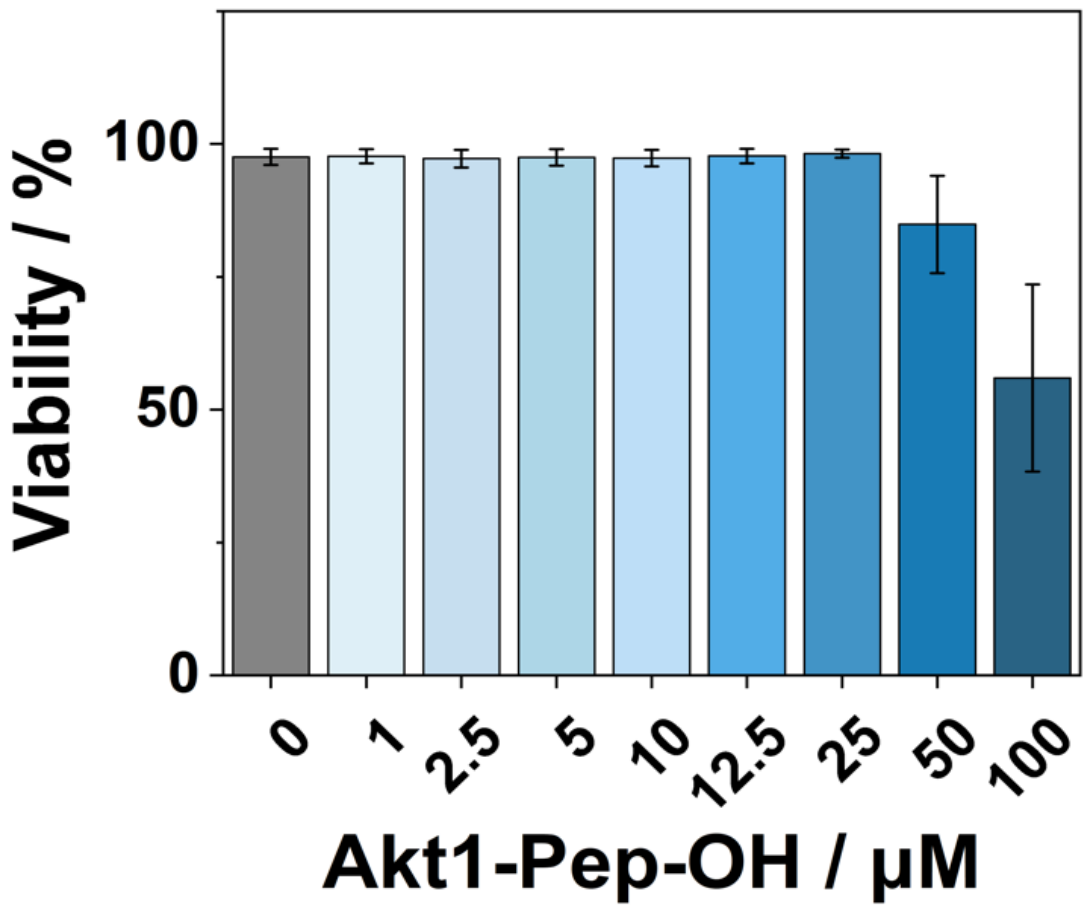

Figure S13. Viability test by flow cytometry. Cytotoxicity profile of the **Akt1-Pep-OH** biosensor in mouse primary T cells after 24 h incubation at increasing concentrations (0–100 μM), measured by flow cytometry. Data are presented as mean ± s.d. from three independent experiments.

2.5. Biosensor cell uptake

Before individual *in celullo* assays, we examined the kinetics of sensor uptake in the Clariostar plate reader. Real-time fluorescence measurements of the **Akt1-Pep1-OH** biosensor (25 μM) in mouse isolated T cells, were taken every 5 min and normalized against untreated cells (average from *n*=3). Cellular uptake kinetics were analyzed by fitting uptake data as a function of time using nonlinear regression in Origin (OriginLab, Northampton, MA, USA). The data were fitted to a one-phase association exponential model, where *y* represents the increase of the biosensor fluorescent emission compared to the non-treated cells.

The results revealed rapid intracellular first-order kinetics accumulation, with a time constant of 13.17 min, reaching ~70% of the maximal fluorescence min and plateauing by ~30 min (Fig. S12). According to these results, 30 min saturation intensity incubating time was fixed for subsequent *in cellulo* assays.

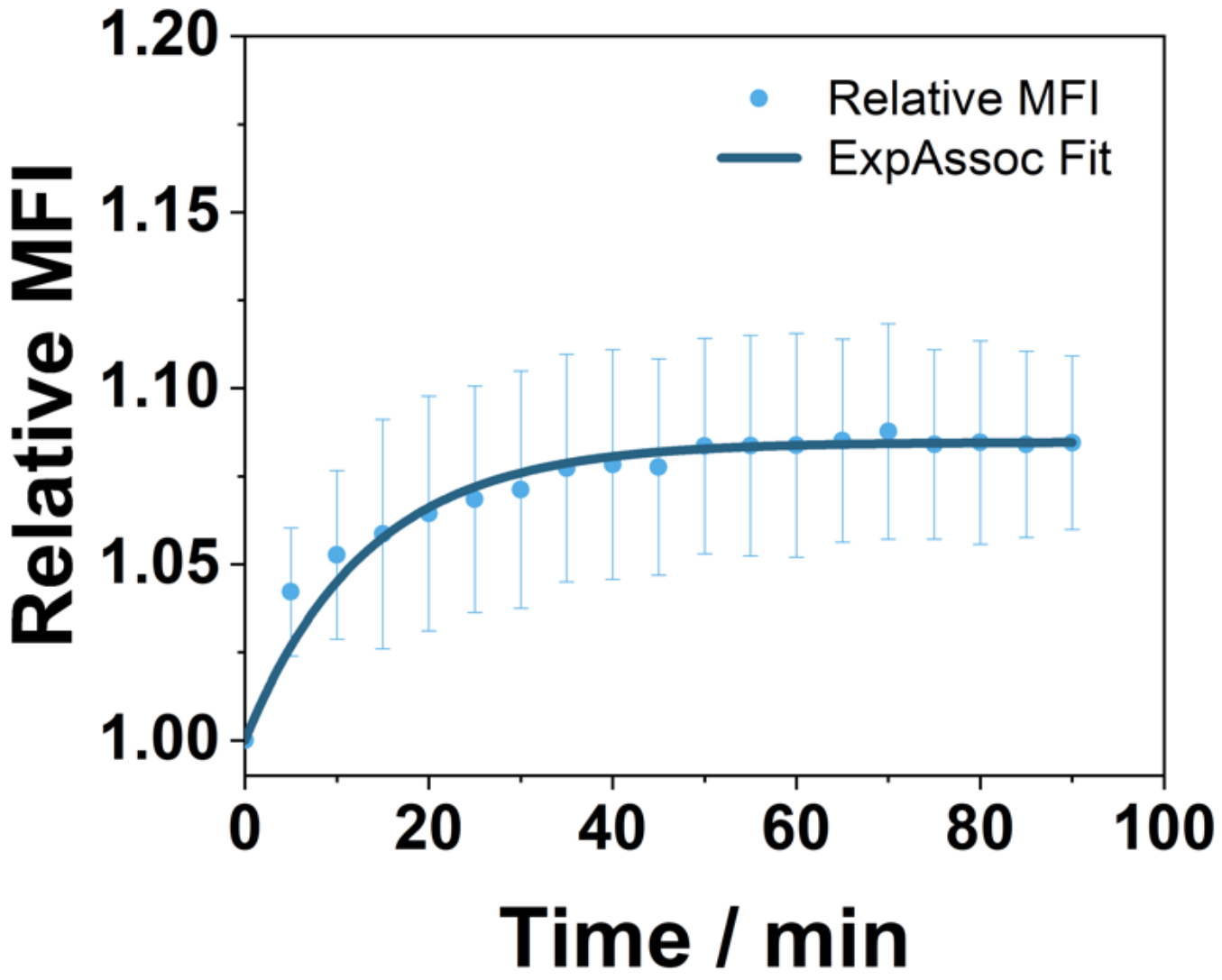

Figure S14. Time-dependent cellular uptake kinetics. Cells were incubated with the **Akt1-Pep1-OH** biosensor (25 µM), and uptake was measured as normalized biosensor emission relative to untreated cells over time. Data are presented as mean ± SD (n = 3). The solid red line represents a nonlinear fit to a one-phase exponential association model, indicating a saturable uptake process.

2.6. Additional imaging figures

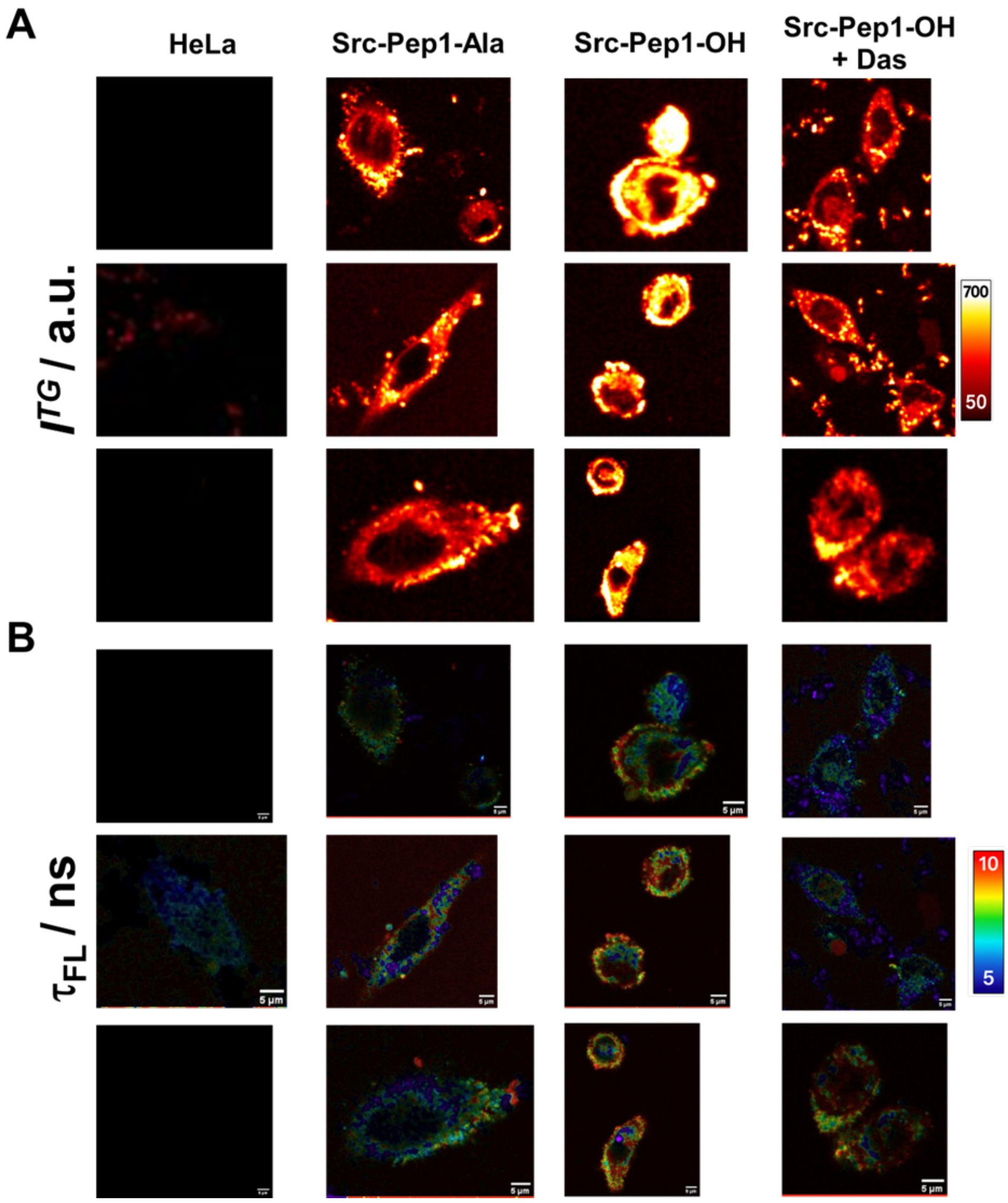

Figure S15. Additional TG-intensity (A) and τ_{FL} imaging (B) of **Src-Pep1-OH/Ala** peptides in HeLa cells. i) Untreated HeLa cells; ii) cells incubated with Src-Pep1-KU530-Ala; iii) cells incubated with **Src-Pep1-OH**; iv) cells pretreated with 40 nM Das and incubated with **Src-Pep1-OH**. The peptide concentration was 5 µM in all cases. Scale bars correspond to 5 µm scale.

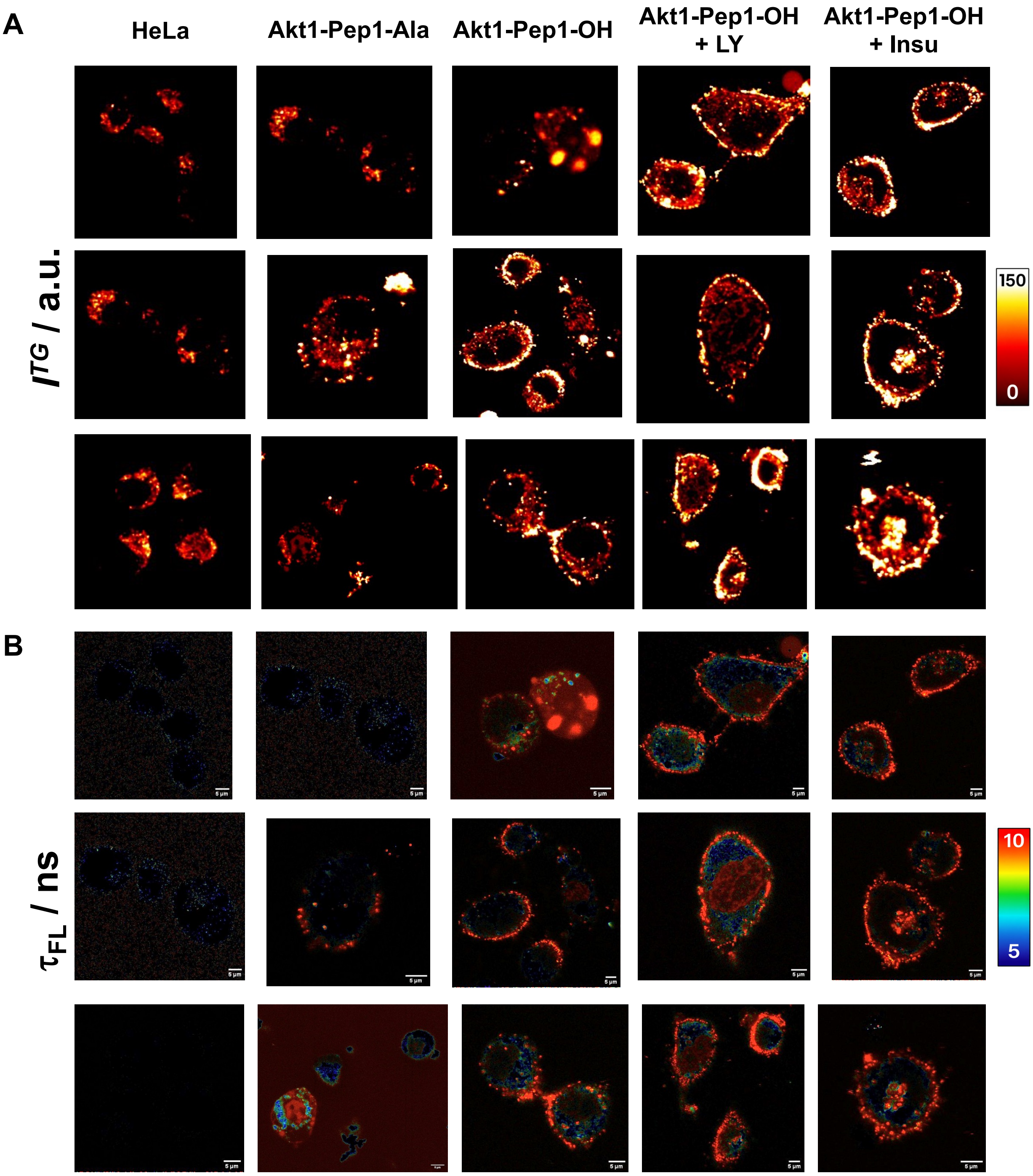

Figure S16. Additional TG-intensity (A) and τ_{FL} imaging (B) of **Akt1-Pep1-OH/Ala** peptides in HeLa cells. i) Untreated HeLa cells; ii) cells incubated with **Akt1-Pep1-Ala**; iii) cells incubated with **Akt1-Pep1-OH**; iv) cells pretreated with 10 µM LY294002 and incubated with **Akt1-Pep1-OH**. The peptide concentration was 5 µM in all cases. Scale bars correspond to 5 µm scale.

3. References

[1] E. Lukovic, J. A. Gonzalez-Vera, B. Imperiali, Recognition-domain focused chemosensors: Versatile and efficient reporters of protein kinase activity, *J. Am. Chem. Soc.* **2008**, *130*, 12821–12827.

[2] M. Peyressatre, A. Laure, M. Pellerano, H. Boukhaddaoui, I. Soussi, M. C. Morris, Fluorescent Biosensor of CDK5 Kinase Activity in Glioblastoma Cell Extracts and Living Cells, *Biotechnol. J.* **2020**, *15*, 1900474.

[3] P. Pracht, F. Bohle, S. Grimme, Automated exploration of the low-energy chemical space with fast quantum chemical methods, *Phys. Chem. Chem. Phys.* **2020**, *22*, 7169–7192.

[4] S. Spicher, S. Grimme, Robust Atomistic Modeling of Materials, Organometallic, and Biochemical Systems, *Angew. Chem. Int. Ed.* **2020**, *59*, 15665–15673.

[5] M. J. Frisch, G. W. Trucks, H. B. Schlegel, G. E. Scuseria, M. A. Robb, J. R. Cheeseman, G. Scalmani, V. Barone, G. A. Petersson, H. Nakatsuji, X. Li, M. Caricato, A. V. Marenich, J. Bloino, B. G. Janesko, R. Gomperts, B. Mennucci, H. P. Hratchian, J. V. Ortiz, A. F. Izmaylov, J. L. Sonnenberg, Williams, F. Ding, F. Lipparini, F. Egidi, J. Goings, B. Peng, A. Petrone, T. Henderson, D. Ranasinghe, V. G. Zakrzewski, J. Gao, N. Rega, G. Zheng, W. Liang, M. Hada, M. Ehara, K. Toyota, R. Fukuda, J. Hasegawa, M. Ishida, T. Nakajima, Y. Honda, O. Kitao, H. Nakai, T. Vreven, K. Throssell, J. A. Montgomery Jr., J. E. Peralta, F. Ogliaro, M. J. Bearpark, J. J. Heyd, E. N. Brothers, K. N. Kudin, V. N. Staroverov, T. A. Keith, R. Kobayashi, J. Normand, K. Raghavachari, A. P. Rendell, J. C. Burant, S. S. Iyengar, J. Tomasi, M. Cossi, J. M. Millam, M. Klene, C. Adamo, R. Cammi, J. W. Ochterski, R. L. Martin, K. Morokuma, O. Farkas, J. B. Foresman, D. J. Fox, Gaussian 16 Rev. C.01, Wallingford, CT, **2016**.

[6] J.-D. Chai, M. Head-Gordon, Long-range corrected hybrid density functionals with damped atom–atom dispersion corrections, *Phys. Chem. Chem. Phys.* **2008**, *10*, 6615–6620.

[7] M. Uhlén, L. Fagerberg, B. M. Hallström, C. Lindskog, P. Oksvold, A. Mardinoglu, Å. Sivertsson, C. Kampf, E. Sjöstedt, A. Asplund, I. Olsson, K. Edlund, E. Lundberg, S. Navani, C. A.-K. Szigyarto, J. Odeberg, D. Djureinovic, J. O. Takanen, S. Hober, T. Alm, P.-H. Edqvist, H. Berling, H. Tegel, J. Mulder, J. Rockberg, P. Nilsson, J. M. Schwenk, M. Hamsten, K. von Feilitzen, M. Forsberg, L. Persson, F. Johansson, M. Zwahlen, G. von Heijne, J. Nielsen, F. Pontén, Tissue-based map of the human proteome, *Science* **2015**, *347*, 1260419.

[8] H. Jin, C. Zhang, M. Zwahlen, K. von Feilitzen, M. Karlsson, M. Shi, M. Yuan, X. Song, X. Li, H. Yang, H. Turkez, L. Fagerberg, M. Uhlén, A. Mardinoglu, Systematic transcriptional analysis of human cell lines for gene expression landscape and tumor representation, *Nat. Commun.* **2023**, *14*, 5417.

[9] M. A. Muñoz-Morris, F. Heitz, G. Divita, M. C. Morris, The peptide carrier Pep-1 forms biologically efficient nanoparticle complexes, *Biochem. Biophys. Res. Commun.* **2007**, *355*, 877–882.

[10] J. Schindelin, I. Arganda-Carreras, E. Frise, V. Kaynig, M. Longair, T. Pietzsch, S. Preibisch, C. Rueden, S. Saalfeld, B. Schmid, J.-Y. Tinevez, D. J. White, V. Hartenstein, K. Eliceiri, P. Tomancak, A. Cardona, Fiji: an open-source platform for biological-image analysis, *Nat. Meth.* **2012**, *9*, 676–682.

[11] D. Wu, M. R. Mand, D. R. Veach, L. L. Parker, B. Clarkson, S. J. Kron, A solid-phase Bcr-Abl kinase assay in 96-well hydrogel plates, *Anal. Biochem.* **2008**, *375*, 18–26.

[12] G. Sun, R. J. A. Budde, Substitution Studies of the Second Divalent Metal Cation Requirement of Protein Tyrosine Kinase CSK, *Biochemistry* **1999**, *38*, 5659–5665.

[13] A. Shivalingam, M. A. Izquierdo, A. L. Marois, A. Vyšniauskas, K. Suhling, M. K. Kuimova, R. Vilar, The interactions between a small molecule and G-quadruplexes are visualized by fluorescence lifetime imaging microscopy, *Nat. Commun.* **2015**, *6*, 8178.

[14] M. Rosenberg, K. R. Rostgaard, Z. Liao, A. Ø. Madsen, K. L. Martinez, T. Vosch, B. W. Laursen, Design, synthesis, and time-gated cell imaging of carbon-bridged triangulenium dyes with long fluorescence lifetime and red emission, *Chem. Sci.* **2018**, *9*, 3122–3130.

[15] B. W. Lewis, N. Bisballe, M. Santella, P. A. Summers, J.-B. Vannier, M. K. Kuimova, B. W. Laursen, R. Vilar, Assessing The Key Photophysical Properties of Triangulenium Dyes for DNA Binding by Alteration of the Fluorescent Core, *Chem. A Eur. J.* **2021**, *27*, 2523–2536.

[16] C. Viedma-Barba, E. M. Ortega-Naranjo, F. Movilla, A. Reinoso, M. Andrades-Amate, P. Peñalver, J. C. Morales, J. A. González-Vera, M. Gutiérrez-Rodríguez, A. Orte, Microwave-assisted synthesis of new triangulenium dyes for lifetime imaging microscopy (FLIM), *Sci. Rep.* **2026**, *16*, 1925.

[17] J. Zhang, S. Mehta, J. Zhang, Protocol for reading and imaging live-cell PKA activity using ExRai-AKAR2, *STAR Protocols* **2022**, *3*, 101071.

4. Annex – Additional Computational Tables

Table S3. Relative energies of the most stable conformations (< 6 kcal/mol) for the **Akt1-OH** peptide obtained with the GFN-FF force field implemented in CREST in aqueous medium.

Conformer	ΔE (kcal/mol)
1	0.000
2	1.151
3	1.294
4	1.558
5	3.228
6	3.419
7	3.577
8	3.699
9	3.798
10	3.850
11	4.046
12	4.491
13	4.502
14	4.552
15	4.570
16	4.661
17	4.661
18	4.671
19	4.709
20	5.219
21	5.244
22	5.419
23	5.462
24	5.463
25	5.485
26	5.521
27	5.676
28	5.905

Table S4. Relative energies of the most stable conformations (< 6 kcal/mol) for the **Akt1-P** peptide obtained with the GFN-FF force field implemented in CREST in aqueous medium.

Conformer	ΔE (kcal/mol)	Conformer	ΔE (kcal/mol)	Conformer	ΔE (kcal/mol)
1	0.000	41	3.236	81	5.420
2	0.064	42	3.276	82	5.433
3	0.353	43	3.396	83	5.458
4	0.456	44	3.490	84	5.484
5	0.592	45	3.537	85	5.494
6	0.649	46	3.546	86	5.496
7	0.687	47	3.612	87	5.568
8	0.752	48	3.689	88	5.571
9	0.774	49	3.792	89	5.574
10	0.780	50	3.881	90	5.601
11	0.934	51	3.883	91	5.610
12	1.243	52	4.063	92	5.661
13	1.375	53	4.091	93	5.667
14	1.541	54	4.225	94	5.671
15	1.621	55	4.261	95	5.676
16	1.701	56	4.445	96	5.690
17	1.744	57	4.666	97	5.690
18	2.025	58	4.669	98	5.693
19	2.026	59	4.674	99	5.755
20	2.106	60	4.785	100	5.768
21	2.238	61	4.813	101	5.778
22	2.324	62	4.835	102	5.797
23	2.357	63	4.850	103	5.798
24	2.405	64	4.866	104	5.813
25	2.438	65	4.916	105	5.814
26	2.503	66	5.015	106	5.848
27	2.587	67	5.069	107	5.854
28	2.599	68	5.088	108	5.883
29	2.639	69	5.127	109	5.908
30	2.743	70	5.140	110	5.923
31	2.852	71	5.155	111	5.942
32	2.857	72	5.241		
33	2.925	73	5.255		
34	2.995	74	5.309		
35	3.033	75	5.318		
36	3.049	76	5.320		
37	3.059	77	5.340		
38	3.162	78	5.359		
39	3.177	79	5.388		
40	3.206	80	5.399		

Table S5. Relative energies of the most stable conformations (< 6 kcal/mol) for the **Src-OH** peptide obtained with the GFN-FF force field implemented in CREST in aqueous medium.

Conformer	ΔE (kcal/mol)	Conf.	ΔE (kcal/mol)	Conf.	ΔE (kcal/mol)	Conf.	ΔE (kcal/mol)
1	0.000	41	4.338	81	5.122	121	5.865
2	0.334	42	4.429	82	5.145	122	5.866
3	1.242	43	4.493	83	5.165	123	5.868
4	1.641	44	4.533	84	5.182	124	5.911
5	1.991	45	4.558	85	5.183	125	5.917
6	2.062	46	4.560	86	5.222	126	5.933
7	2.166	47	4.593	87	5.226	127	5.942
8	2.188	48	4.625	88	5.231	128	5.951
9	2.281	49	4.643	89	5.265		
10	2.313	50	4.653	90	5.272		
11	2.409	51	4.684	91	5.290		
12	2.475	52	4.735	92	5.293		
13	2.494	53	4.737	93	5.326		
14	2.560	54	4.756	94	5.346		
15	2.600	55	4.763	95	5.373		
16	2.683	56	4.767	96	5.389		
17	2.729	57	4.795	97	5.399		
18	2.739	58	4.807	98	5.455		
19	2.811	59	4.827	99	5.455		
20	2.861	60	4.828	100	5.459		
21	2.911	61	4.842	101	5.512		
22	3.119	62	4.857	102	5.518		
23	3.197	63	4.860	103	5.523		
24	3.216	64	4.872	104	5.546		
25	3.249	65	4.874	105	5.565		
26	3.317	66	4.915	106	5.634		
27	3.499	67	4.924	107	5.661		
28	3.567	68	4.880	108	5.670		
29	3.622	69	4.927	109	5.671		
30	3.645	70	4.931	110	5.687		
31	3.744	71	4.937	111	5.694		
32	3.808	72	4.976	112	5.702		
33	3.857	73	5.008	113	5.717		
34	3.925	74	5.013	114	5.750		
35	4.146	75	5.046	115	5.761		
36	4.235	76	5.058	116	5.776		
37	4.314	77	5.068	117	5.787		
38	4.323	78	5.081	118	5.794		
39	4.335	79	5.110	119	5.814		
40	4.335	80	5.116	120	5.833		

Table S6. Relative energies of the most stable conformations (< 6 kcal/mol) for the **Src-P** peptide obtained with the GFN-FF force field implemented in CREST in aqueous medium.

Conformer	ΔE (kcal/mol)	Conf.	ΔE (kcal/mol)	Conf.	ΔE (kcal/mol)	Conf.	ΔE (kcal/mol)
1	0.000	42	2.549	83	3.391	124	3.993
2	0.092	43	2.583	84	3.412	125	3.997
3	0.395	44	2.597	85	3.417	126	4.005
4	0.530	45	2.605	86	3.432	127	4.023
5	0.598	46	2.660	87	3.562	128	4.028
6	0.652	47	2.668	88	3.588	129	4.029
7	0.745	48	2.755	89	3.601	130	4.050
8	0.872	49	2.767	90	3.642	131	4.062
9	0.886	50	2.814	91	3.646	132	4.072
10	1.011	51	2.832	92	3.647	133	4.086
11	1.101	52	2.863	93	3.667	134	4.091
12	1.157	53	2.868	94	3.689	135	4.129
13	1.233	54	2.885	95	3.690	136	4.130
14	1.457	55	2.915	96	3.690	137	4.147
15	1.474	56	2.932	97	3.701	138	4.167
16	1.496	57	2.935	98	3.702	139	4.173
17	1.514	58	2.938	99	3.693	140	4.197
18	1.530	59	2.949	100	3.739	141	4.220
19	1.578	60	2.951	101	3.769	142	4.246
20	1.675	61	2.961	102	3.783	143	4.223
21	1.798	62	3.012	103	3.784	144	4.256
22	1.809	63	3.014	104	3.789	145	4.257
23	1.885	64	3.027	105	3.795	146	4.265
24	2.016	65	3.044	106	3.803	147	4.290
25	2.025	66	3.049	107	3.833	148	4.317
26	2.057	67	3.098	108	3.843	149	4.333
27	2.080	68	3.105	109	3.862	150	4.333
28	2.134	69	3.141	110	3.862	151	4.359
29	2.138	70	3.144	111	3.875	152	4.378
30	2.164	71	3.168	112	3.878	153	4.383
31	2.179	72	3.189	113	3.887	154	4.388
32	2.202	73	3.223	114	3.892	155	4.391
33	2.207	74	3.264	115	3.899	156	4.394
34	2.240	75	3.269	116	3.906	157	4.396
35	2.266	76	3.274	117	3.923	158	4.407
36	2.305	77	3.288	118	3.931	159	4.421
37	2.350	78	3.292	119	3.943	160	4.425
38	2.416	79	3.350	120	3.949	161	4.449
39	2.528	80	3.351	121	3.952	162	4.470
40	2.542	81	3.369	122	3.979	163	4.477
41	2.544	82	3.381	123	3.989	164	4.500
165	4.518	211	4.897	257	5.502	303	5.893
166	4.519	212	4.930	258	5.511	304	5.896

Conformer	ΔE (kcal/mol)	Conf.	ΔE (kcal/mol)	Conf.	ΔE (kcal/mol)	Conf.	ΔE (kcal/mol)
167	4.530	213	4.939	259	5.514	305	5.908
168	4.555	214	4.962	260	5.517	306	5.921
169	4.557	215	4.966	261	5.529	307	5.940
170	4.558	216	4.976	262	5.555	308	5.942
171	4.563	217	4.989	263	5.576	309	5.945
172	4.573	218	4.990	264	5.580	310	5.947
173	4.575	219	5.013	265	5.584	311	5.958
174	4.576	220	5.029	266	5.587	312	5.970
175	4.577	221	5.041	267	5.592	313	5.971
176	4.591	222	5.052	268	5.598	314	5.983
177	4.592	223	5.064	269	5.625		
178	4.596	224	5.086	270	5.625		
179	4.608	225	5.090	271	5.632		
180	4.613	226	5.104	272	5.659		
181	4.652	227	5.107	273	5.668		
182	4.675	228	5.109	274	5.704		
183	4.677	229	5.160	275	5.707		
184	4.680	230	5.165	276	5.709		
185	4.694	231	5.175	277	5.714		
186	4.705	232	5.178	278	5.718		
187	4.713	233	5.178	279	5.721		
188	4.716	234	5.186	280	5.728		
189	4.718	235	5.202	281	5.732		
190	4.721	236	5.211	282	5.733		
191	4.724	237	5.213	283	5.739		
192	4.726	238	5.232	284	5.749		
193	4.734	239	5.240	285	5.758		
194	4.760	240	5.250	286	5.762		
195	4.762	241	5.266	287	5.777		
196	4.772	242	5.294	288	5.789		
197	4.802	243	5.304	289	5.823		
198	4.808	244	5.330	290	5.826		
199	4.819	245	5.340	291	5.827		
200	4.821	246	5.345	292	5.827		
201	4.822	247	5.363	293	5.830		
202	4.824	248	5.372	294	5.840		
203	4.830	249	5.392	295	5.852		
204	4.835	250	5.402	296	5.855		
205	4.851	251	5.411	297	5.858		
206	4.853	252	5.447	298	5.859		
207	4.860	253	5.447	299	5.860		
208	4.861	254	5.482	300	5.871		
209	4.897	255	5.483	301	5.877		
210	4.906	256	5.488	302	5.888		
